# Unveiling the Role of ZnCl_2_ in Enhancing the Photoluminescence Efficiency of Amino-As-Based InAs@ZnSe Quantum Dots

**DOI:** 10.1021/acsnano.5c10371

**Published:** 2025-09-25

**Authors:** Dongxu Zhu, Jordi Llusar, Aswin Asaithambi, Zheming Liu, René Bes, Damien Prieur, Hiba H. Karakkal, Mirko Prato, Sergio Brovelli, Gabriele Saleh, Satyaprakash Panda, Ivan Infante, Luca De Trizio, Liberato Manna

**Affiliations:** † Nanochemistry, 121451 Istituto Italiano di Tecnologia, Via Morego 30, 16163 Genova, Italy; ‡ Materials Characterization, 121451 Istituto Italiano di Tecnologia, Via Morego 30, 16163 Genova, Italy; § Chemistry Facility, 121451 Istituto Italiano di Tecnologia, Via Morego 30, 16163 Genova, Italy; ∥ Department of Physics and Helsinki Institute of Physics, University of Helsinki, P.O. Box 43, FI-00014 Helsinki, Finland; ⊥ HZDR, Institute of Resource Ecology, Bautzner Landstraße 400, 01328 Dresden, Germany; # Rossendorf Beamline (BM20-CRG), European Synchrotron Radiation Facility, Avenue des Martyrs 71, 38043 Grenoble, France; ¶ Dipartimento di Scienza dei Materiali, Università degli Studi di Milano-Bicocca, Via R. Cozzi 55, 20125 Milano, Italy; ∇ BCMaterials, Basque Center for Materials, Applications, and Nanostructures, UPV/EHU Science Park, 48940 Leioa, Spain; ○ Dipartimento di Chimica e Chimica Industriale, Università di Genova, 16146 Genova, Italy; ⧫ Ikerbasque Basque Foundation for Science, 48009 Bilbao, Spain

**Keywords:** InAs quantum dots, core@shell InAs@ZnSe, III−V semiconductor, ZnCl_2_ additive, NIR emission, RoHS compliant

## Abstract

We investigated how ZnCl_2_, employed as an additive in the amino-As-based synthesis of indium arsenide (InAs) quantum dots (QDs), considerably improves the photoluminescence quantum yield (PLQY) of the resulting InAs@ZnSe core@shell QDs. We achieved this by synthesizing and comparing three distinct InAs QD samples and their corresponding core@shell structures: (1) In­(Zn)As QDs (synthesized with ZnCl_2_); (2) standard InAs QDs (std-InAs, made without additives); and (3) std-InAs QDs postsynthesis treated with ZnCl_2_ (Zn–InAs). High PLQY values (∼70%) were attained only with In­(Zn)­As@ZnSe QDs, while std-InAs@ZnSe and Zn–InAs@ZnSe samples exhibited much lower PL efficiencies (10–20%). We also demonstrated that (i) the high PLQY in In­(Zn)­As@ZnSe QDs could not be attributed solely to the presence of an In–Zn–Se interlayer, as this was present in all three samples; (ii) the specific ZnSe shelling procedure had only a minor impact on the final PLQY; and (iii) the PL efficiency was significantly improved only when high amounts of ZnCl_2_ additive (specifically with ZnCl_2_:InCl_3_ precursor ratios over 10:1) were used during the InAs QDs synthesis. These findings were rationalized through density functional theory (DFT) calculations coupled with X-ray absorption spectroscopy measurements. DFT models suggested that std-InAs QDs feature surface trap states, mainly located on the (−1–1–1) facets, thus low PL efficiency even after ZnSe shelling. The use of ZnCl_2_ in the InAs synthesis led to surface Zn incorporation, particularly on the (100) and (−1–1–1) facets, effectively passivating surface traps and, consequently, yielding highly emissive In­(Zn)­As@ZnSe QD systems. In contrast, ZnCl_2_ employed in the postsynthesis treatment of std-InAs QDs resulted only in a limited surface Zn incorporation and in ZnCl_2_ adsorption on the (−1–1–1) facets (i.e., ZnCl_2_ acting as a Z-type ligand), leading to poor passivation of surface traps. Overall, our study demonstrates the critical role of ZnCl_2_ as a synthesis additive in delivering highly emissive amino-As-based InAs@ZnSe QDs.

## Graphical abstract

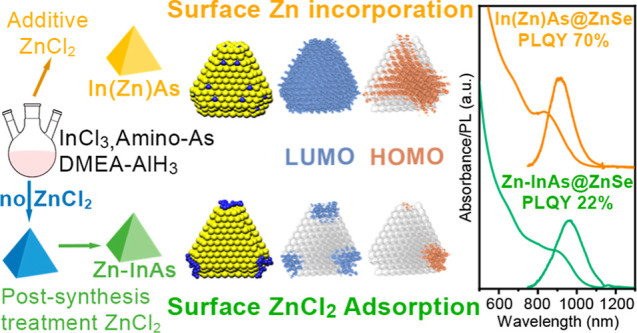

Infrared (IR)-emitting quantum dots (QDs) have attracted significant attention due to their wide-ranging potential applications, including optical communication systems, biological imaging, night and fog visors, objects and food inspection systems, and security cameras.^1–4^ The most well-developed IR-emitting QDs are Hg-based (II–VI)^5^ and Pb-based (IV–VI) QDs.^4^ However, these materials are restricted in electrical and electronic equipment under the European Union’s “Restriction of Hazardous Substances” (RoHS) directives, driving the search for alternative QD materials. Promising alternatives are primarily limited to silver chalcogenides,^6^ silver-^7^ and copper-based I–III–VI semiconductors,^8^ and III–V QDs, such as indium arsenide (InAs)^9^ and indium antimonide (InSb).^10^ Among these, InAs QDs exhibit a tunable optical bandgap covering the visible spectrum to approximately 1700 nm,^11–16^ making them strong candidates for commercial IR applications.

A key challenge in the synthesis of InAs QDs is the limited availability of suitable arsenic precursors, with well-developed syntheses employing mostly tris­(trimethylsilyl)- and tris­(trimethylgermyl) arsine (TMS-As).^9,17–21^ However, these arsenic precursors are toxic, highly reactive, costly, and commercially limited. To address these issues, alternative cheaper and “safer” arsenic precursors have been tested:^16,22–26^ among them, tris­(dimethylamino)­arsine (amino-As), in conjunction with a reducing agent, is the most promising one.^27,28^ Indeed, various synthesis approaches based on amino-As and different reducing agents (e.g., In­(I) halides, tris­(dimethylamino)­phosphine, diisobutylaluminium hydride, 1,1,3,3,5,5-hexamethyltrisiloxane, etc.) have been shown to produce amino-As-based InAs QDs with excitonic absorption peaks ranging from the visible region down to 1700 nm in the IR.^11,13,15,27–33^ Moreover, in terms of photoluminescence (PL) efficiency, InAs QDs synthesized with amino-As, specifically InAs@ZnSe core@shell QDs, have achieved PL quantum yield (QY) values as high as 70% at wavelengths up to ∼950 nm.^30,33^ These results were obtained only when ZnCl_2_ was used as an additive during the synthesis of InAs QDs prior to overgrowth of the ZnSe shell.

To date, the exact role of ZnCl_2_ in the overall synthesis process has remained unclear. In a previous study of ours, we observed that the addition of ZnCl_2_ to the synthesis of InAs QDs, based on amino-As and alane *N*,*N*-dimethylethylamine (DMEA-AlH_3_) as its reducing agent, could slightly improve the control over the QDs size distribution and the PL efficiency of the QDs.^33^ These improvements were tentatively ascribed to the possibility that ZnCl_2_ acts as a Z-type ligand, passivating the surface of InAs QDs.^33^ Moreover, the “one-pot” overgrowth of a ZnSe shell (i.e., the addition of Zn and Se precursors to the crude QDs reaction mixture) onto InAs QDs prepared using ZnCl_2_ as an additive resulted in InAs@ZnSe core@shell structures featuring an In–Zn–Se interlayer between the InAs core and the ZnSe shell.^30^ This interlayer was found to mitigate the lattice mismatch between the two materials (6.4%), thereby leading to high PLQY values. Yet, it remained still uncertain whether Zn ions are just adsorbed on the surface of InAs QDs (i.e., as ZnCl_2_ species) or are incorporated in their lattice and how they contribute to the record PLQY values observed in the resulting InAs@ZnSe QDs.

To address these open questions, in the present work, we prepared three different types of InAs QD samples, namely: (1) InAs QDs synthesized with ZnCl_2_ as an additive, referred to as In­(Zn)­As; (2) “standard” InAs QDs made without additives (std-InAs); (3) std-InAs QDs subjected to a postsynthesis treatment with ZnCl_2_ (Zn–InAs), achieving a Zn content similar to the one measured for In­(Zn)­As. Each type of QD was subsequently overcoated with a ZnSe shell using an optimized “one-pot” method described in our previous study (Scheme 1).^30^

**Scheme 1:**
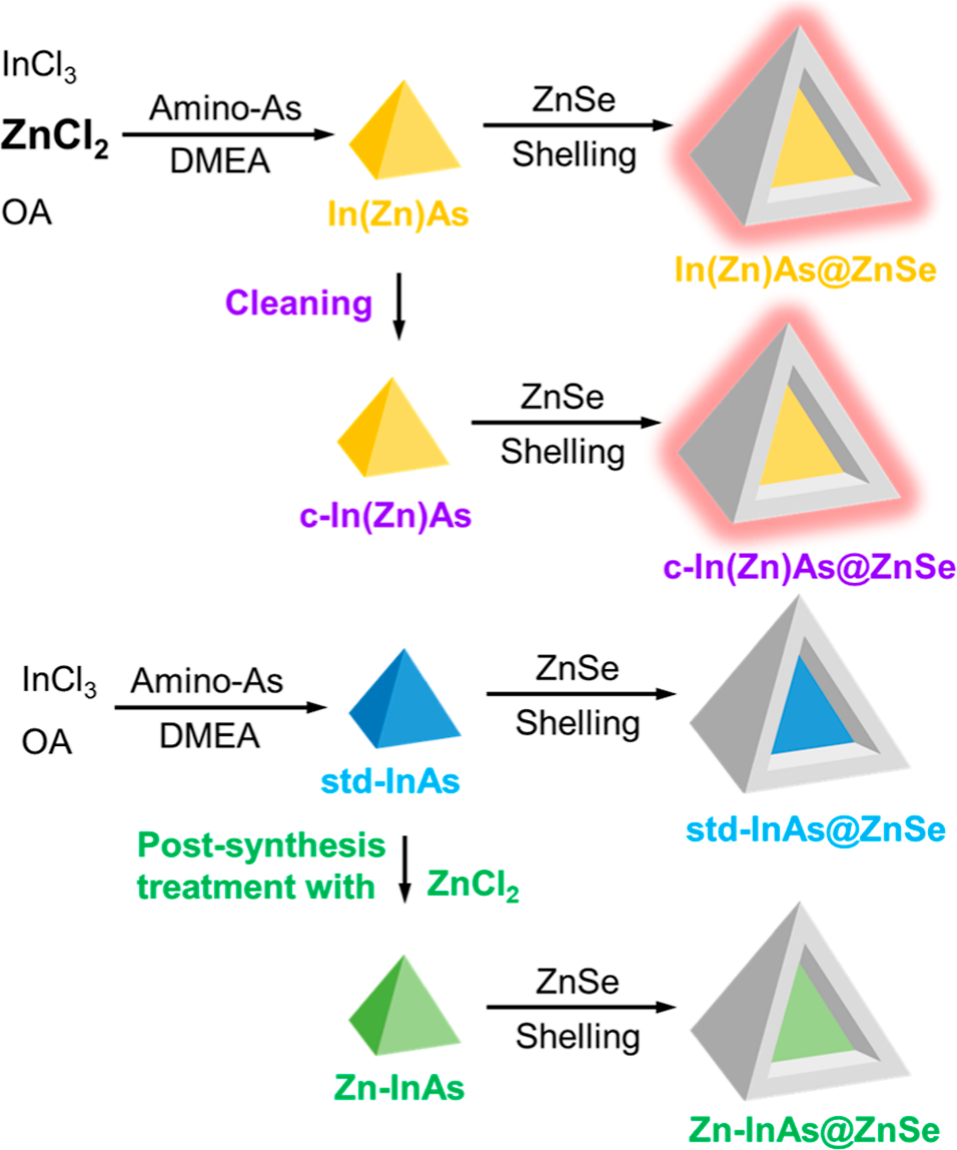
Schematic Representation of the Synthesis of InAs QDs Either Made with ZnCl_2_ as an Additive (In­(Zn)­As) or without Additives (“Standard” InAs, Std-InAs) and of InAs QDs Prepared via Postsynthesis Treatment of Std-InAs QDs with ZnCl_2_ (Zn–InAs) and Their Corresponding InAs@ZnSe Core@Shell Structures^a^

a c-In­(Zn)­As QDs are In­(Zn)As QDs subjected to cleaning before their subsequent ZnSe shell growth.

Our experiments revealed that only when employing ZnCl_2_ in the synthesis, and not in the postsynthesis treatment of InAs QDs, it was possible to achieve an effective passivation of surface trap states. As a result, upon ZnSe shell growth, the In­(Zn)As QDs reached a PLQY as high as ∼70%, whereas std-InAs and Zn–InAs QDs featured PL efficiencies of only 10% and 22%, respectively. Structural characterizations combined with ad-hoc control experiments indicated that (i) the In–Zn–Se interlayer, despite being essential in order for improving the PLQY of In­(Zn)­As@ZnSe QDs, was not the only factor responsible for the high PLQY of this system, as such interlayer was present in all three systems; (ii) high PLQY could be achieved through both a “one-pot” procedure and a “two-pot” procedure (i.e., ZnSe growth on In­(Zn)As QDs washed prior to shelling, c-In­(Zn)­As, Scheme 1), indicating that PL efficiency was not linked to the shelling method but rather to the use of ZnCl_2_, whose amount should be at least 10 times that of the In precursor, during InAs QD synthesis.

We also performed density functional theory (DFT) calculations and X-ray absorption spectroscopy (XAS) measurements, which revealed that Zn was incorporated at the surface of In­(Zn)As QDs with a preference for the (100) and (−1–1–1) facets. Such Zn incorporation, particularly at the (−1–1–1) facets, results in efficient surface trap passivation, ultimately leading to high PLQY values in the corresponding In­(Zn)­As@ZnSe QDs. Conversely, ZnCl_2_ postsynthesis treatment resulted in limited surface Zn incorporation and ZnCl_2_ adsorption onto the (−1–1–1) facets of Zn–InAs QDs. Such Zn distribution yields Zn–InAs QDs with only poor surface trap passivation, similar to the case of std-InAs QDs, which is likely partially retained upon ZnSe shell growth, ultimately resulting in std-InAs@ZnSe and Zn–InAs@ZnSe QDs with low PLQY values.

## Results and Discussion

### Synthesis and Characterization of “Core” InAs QDs

We synthesized In­(Zn)As QDs using our previously reported method, which employs InCl_3_, amino-As, ZnCl_2_, oleylamine (OA), and DMEA-AlH_3_.^30,33^ The InCl_3_:ZnCl_2_ precursor ratio was fixed to 20:1, and the reaction temperature was maintained at 300 °C (see the Materials and Methods section for details). Std-InAs QDs were synthesized following the same approach but without the use of ZnCl_2_ as an additive. To prepare Zn–InAs QDs, we first synthesized std-InAs QDs and subsequently treated them postsynthesis by adding 20 equiv of ZnCl_2_ (corresponding to a ZnCl_2_:InCl_3_ ratio of 20:1) to the crude std-InAs QDs reaction mixture. The final mixture was then heated to 280 °C for 3 h to achieve a Zn content similar to that of the In­(Zn)As QDs (vide infra; see Supporting Information, for details).

All three samples consisted of QDs with the expected cubic InAs zinc-blende structure (Figure S1), a similar size (approximately 3 nm, Figure S2), and an In-rich surface termination (In/As atomic ratios of ∼1.1, Table 1), as commonly reported for these QDs.^30–33^ The postsynthesis ZnCl_2_ treatment used to prepare Zn–InAs QDs (see the Materials and Methods section and Figures S3 and S4a) resulted in a Zn content comparable to that of In­(Zn)As QDs, with Zn/As atomic ratios of 0.15 and 0.12, respectively (measured via ICP-OES, see Table 1). X-ray photoelectron spectroscopy (XPS) analysis of the samples revealed that (i) In was present in the same chemical state across all samples. The In 3d_5/2_ peak position (Figure 1a) was consistently located at (444.4 ± 0.2) eV, indicating no variation in the chemical state of indium across the three samples. (ii) Similarly, the As 3d_5/2_ peaks were nearly identical and centered at (40.7 ± 0.2) eV in all cases (Figure 1b). Notably, no In or As oxides were detected in the XPS spectra (Figure 1a,b). (iii) The Zn 2p spectrum of Zn–InAs QDs displayed two distinct chemical states. The main Zn 2p_3/2_ component appeared at (1021.8 ± 0.2) eV, the same binding energy as that found in In­(Zn)­As QDs (Figure 3c). A secondary component, located at a higher binding energy of (1022.6 ± 0.2) eV, was in line with the Zn signal measured for ZnCl_2_ (i.e., 1022.4 ± 0.2 eV, Figure S4b) and was therefore attributed to the latter. A control experiment, in which Zn–InAs QDs were deliberately oxidized by prolonged exposure to air, confirmed this attribution, ruling out the possibility that the second Zn component originated from ZnO-related species (Figure S5).

**Table 1:** Atomic Ratios, Size, and Optical Data of In­(Zn)­As, Std-InAs, and Zn–InAs QD Samples

sample	In/As ratio^a^	Zn/As ratio^a^	size (nm)	Abs peak position (nm)	HWHM of Abs. (meV)	PLQY (%)	PL lifetime at RT (ns)
In(Zn)As	1.07	0.12	3	865	104	2 ± 1	τ_1_ = 4 ns (29%), τ_2_ = 24 ns (71%)
std-InAs	1.11	0	3	845	111	<0.5	τ = 2
Zn–InAs	1.07	0.15	3	870	113	<0.5	τ = 2

a The atomic ratios were measured via ICP-OES analysis.

**Figure 1:**
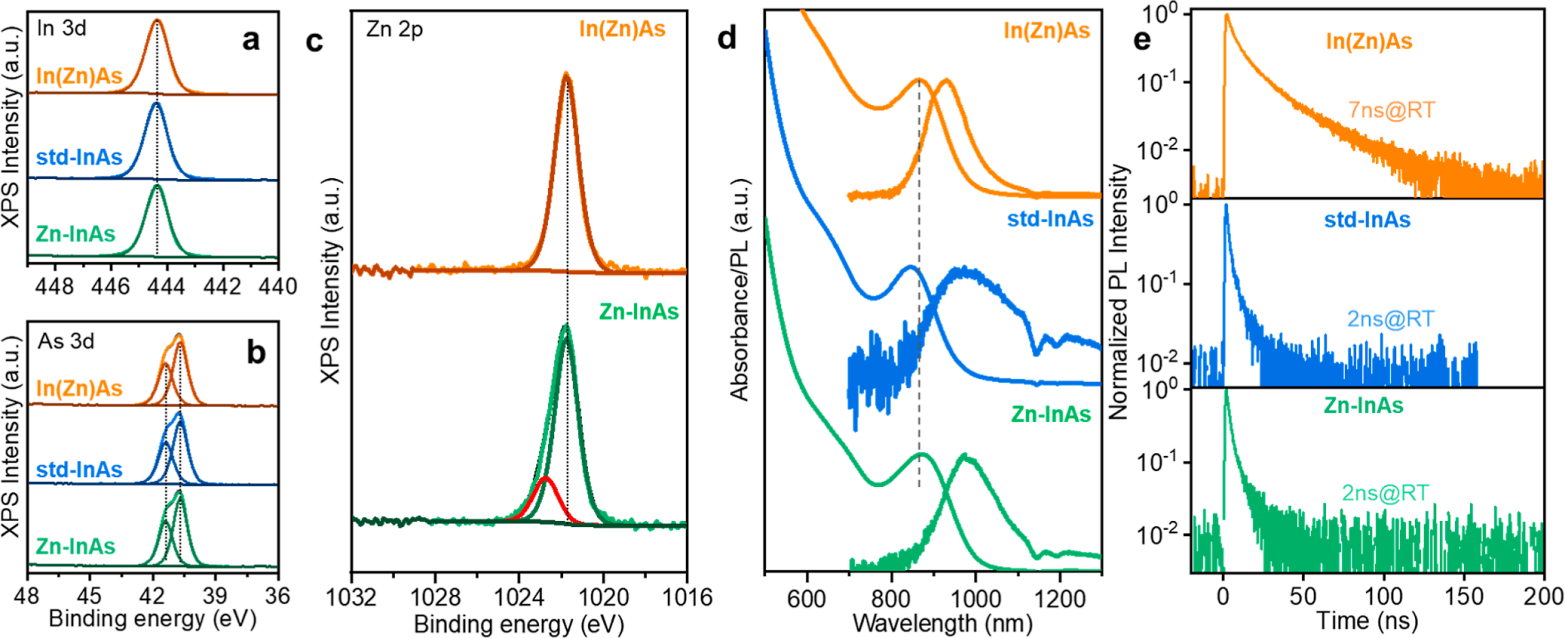
(a–c) XPS analysis of the InAs QD samples: (a) In 3d_5/2_, (b) As 3d, and (c) Zn 2p_3/2_ spectra. (d) Optical absorption and PL spectra and (e) PL decay traces acquired at room temperature for In­(Zn)­As, InAs, and Zn–InAs QDs.

All three samples exhibited an excitonic absorption peak at around 860 nm with a half-width at half-maximum (HWHM) of ∼110 meV (Figure 1d and Table 1). Notably, the ZnCl_2_ treatment applied to std-InAs to obtain Zn–InAs QDs resulted in a slight red shift of their optical absorption peak from 845 to 870 nm while maintaining the original HWHM (around 111 meV, see Figure 1d and Table 1). Despite having similar absorption profiles, the three samples exhibited significant differences in their PL properties (Table 1 and Figure 1d): (a) the PL spectrum of In­(Zn)As QDs was the only one mirroring the respective excitonic absorption, whereas the others were broader and had a larger Stokes shift; (b) In­(Zn)As QDs had the highest PLQY at ∼2%. This is consistent with time-resolved PL measurements (Figure 1e) that revealed similar fast decay kinetics for the std-InAs and Zn–InAs QDs (with lifetimes τ, around 2 ns, Table 1), indicative of similar recombination dominated by carrier trapping. In contrast, the In­(Zn)­As QDs featured a slower decay with an initial τ_1_ = 4 ns component, indicating the presence of residual surface trapping, followed by a longer-lived (τ_2_ = 24 ns) tail that accounted for the majority of the signal (∼70%, Table 1).

### Synthesis and Characterization of InAs@ZnSe Core@Shell QDs

To improve the PLQY of the three InAs QD systems (after quenching the QDs growth or completing the postsynthesis treatment in the case of Zn–InAs QDs), we performed our optimized “one-pot” ZnSe shelling procedure to prepare the corresponding InAs@ZnSe core@shell heterostructures.^30^ Specifically, this procedure involves adding trioctylphosphine (TOP)–Se and ZnCl_2_–OA solutions to the crude reaction mixture at 90 °C, ensuring a total In:As:Zn:Se feed ratio of 1:1:34:37.5 in all cases. The reaction mixture was then heated to 310 °C for 2 h (see the Materials and Methods section for details). In all three cases, the resulting core@shell QDs featured a shell thickness of approximately 6–7 monolayers (ML) with a cubic zinc-blende structure without secondary phases (see Table 2 and Figure 2d). The shell thickness was estimated from the QD size measured via transmission electron microscopy (TEM) analysis, combined with elemental analysis performed by inductively coupled plasma-optical emission spectroscopy (ICP-OES) and by resorting to a structural model already reported in our previous work (see Figure 2a–c, Tables 1 and 2).^30^ Specifically, the structural model consisted of a 3 nm tetrahedral InAs core surrounded by a shell of variable thickness (see ref 30).

**Table 2:** Atomic Ratios, Size, ZnSe Thickness, and Optical Data of In­(Zn)­As@ZnSe, Std-InAs@ZnSe, and Zn–InAs@ZnSe QD Samples

sample	In/As ratio^a^	Zn/As ratio^a^	Zn/Se ratio^a^	size (nm)	ZnSe ML	PLQY (%)	PL lifetime
In(Zn)As@ZnSe	1.84	23.71	1.00	9.3 ± 1.3	7	70 ± 7	τ = 52 ns
std-InAs@ZnSe	2.03	24.85	1.02	9.6 ± 1.4	7	10 ± 1	τ_1_ = 4 ns (6%), τ_2_ = 41 ns (94%)
Zn–InAs@ZnSe	1.76	19.68	1.13	8.4 ± 1.2	6	22 ± 2	τ = 47 ns

a The atomic ratios were measured via ICP-OES analysis.

**Figure 2:**
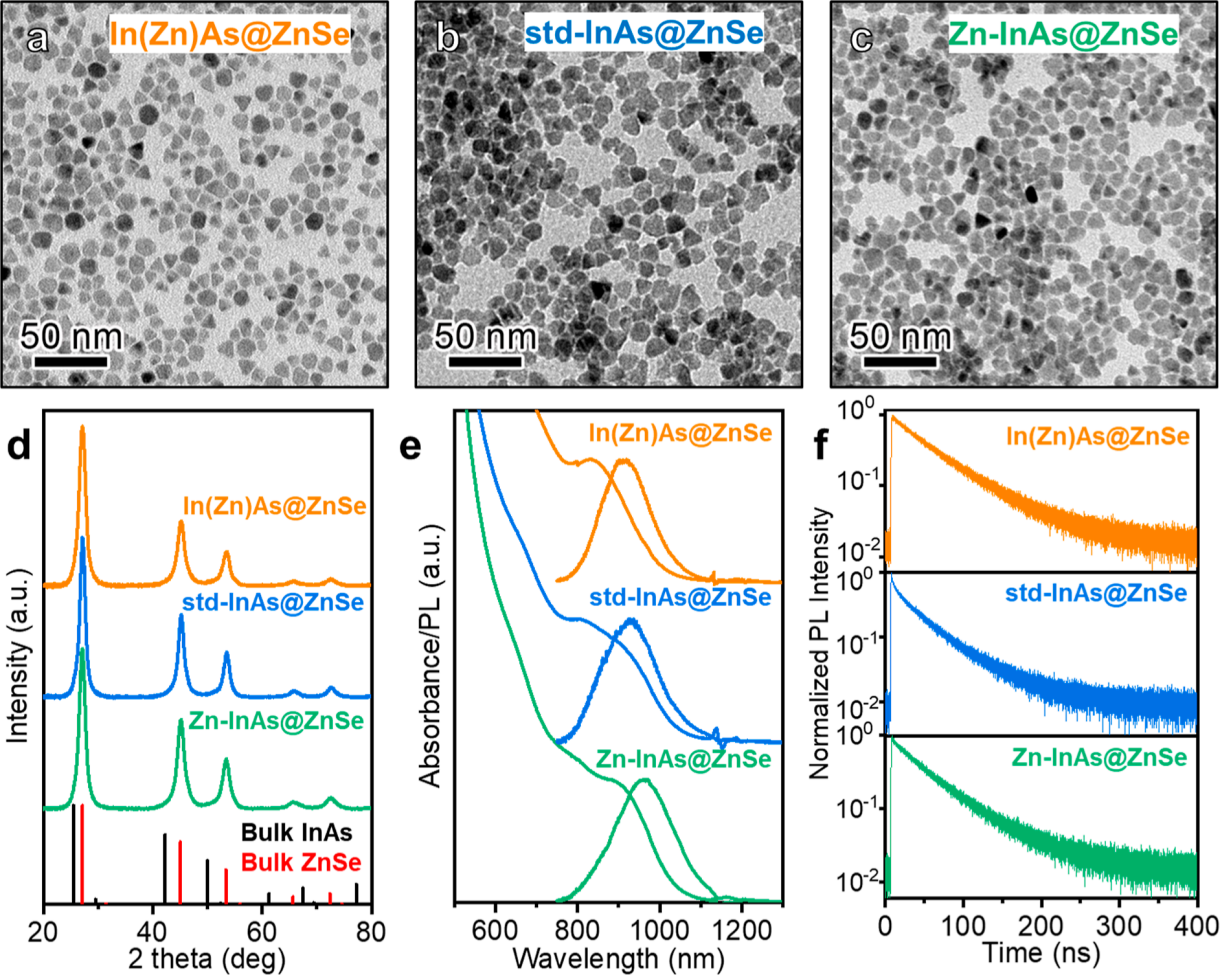
(a–c) TEM micrographs and (d) X-ray diffraction (XRD) patterns of In­(Zn)­As@ZnSe, std-InAs@ZnSe, and Zn–InAs@ZnSe core@shell QDs together with the bulk reflections of InAs (ICSD 98-002-4518) and ZnSe (ICSD 98-007-7092). (e,f) Optical characterization of In­(Zn)­As@ZnSe, std-InAs@ZnSe, and Zn–InAs@ZnSe core@shell QDs: (e) optical absorption and PL spectra and (f) PL decay traces.

All core@shell samples exhibited a distinct PL peak with a similar shape and spectral shift from the respective excitonic absorption. In­(Zn)­As@ZnSe QDs showed a PLQY as high as 70 ± 7%, consistent with our previous study,^30^ whereas std-InAs@ZnSe and Zn–InAs@ZnSe QDs were characterized by significantly lower PLQY values of ∼10% and 22%, respectively (Table 2 and Figure 2e). In all three cases, upon ZnSe shelling, a broadening of the excitonic absorption peak was observed, which was more pronounced in the std-InAs and Zn–InAs samples compared to the In­(Zn)As one (Figures 1d, 2e, and S6). Time-resolved PL measurements were aligned with the PLQY data, revealing double exponential decay dynamics for std-InAs@ZnSe QDs with a residual trapping component accounting for 6% of the signal (Figure 2f and Table 2). In contrast, In­(Zn)­As@ZnSe QDs showed a predominantly single-exponential decay with a longer PL lifetime of 52 ns, indicative of effective defect passivation, and consistent with the high PLQY observed for these QDs. Zn–InAs@ZnSe QDs also showed a predominantly single-exponential decay with a lifetime of 47 ns which is slightly faster than that of In­(Zn)­As@ZnSe QDs.

It is also noteworthy that in all three cases, the In/As ratio increased during the whole ZnSe shelling process, from the initial ∼1.1 value (Table 1), observed in their corresponding core-only counterparts, up to ∼1.7–2 (Tables 2 and S1). This suggests the formation of an In–Zn–Se “interlayer” during the ZnSe shell growth in all the three cases, as detailed in our previous work.^30,33^ We previously hypothesized that the high PLQY of the In­(Zn)­As@ZnSe QDs synthesized using our method could be attributed to such an “interlayer”, localized between the InAs core and the ZnSe shell, which mitigates the high lattice mismatch (6.4%) between InAs and ZnSe.^30,33^ However, according to our new experimental results, we note that (i) the presence of such an “interlayer” is a necessary but not sufficient condition to achieve a high PLQY in InAs@ZnSe QDs; (ii) high PL efficiencies in InAs@ZnSe QDs can only be obtained when ZnCl_2_ is used during the InAs QD synthesis; (iii) post-treatment of InAs QDs with ZnCl_2_ leads to Zn incorporation; however, this is not enough to produce core@shell QDs with optimal PL efficiencies.

We also conducted a set of control experiments (Figure 3a) to assess: (i) whether the reactants and byproducts of the In­(Zn)As QD synthesis, present during the “one-pot” ZnSe shelling process, influence ZnSe shell formation and growth and, consequently, the final PLQY of In­(Zn)­As@ZnSe QDs; (ii) the optimal ZnCl_2_ concentration, if any, required to achieve In­(Zn)­As@ZnSe with high PL efficiency. In a first experiment, the ZnSe shell was grown onto purified In­(Zn)As QDs (c-In­(Zn)­As) rather than being grown in a “one pot” (Figure 3a upper scheme). This approach ensured that the ZnSe shell developed without any influence from the unreacted species remaining from the In­(Zn)As QD synthesis. In the resulting c-In­(Zn)­As@ZnSe QDs, the shell thickness was 7 ML (Figure S7) and an In–Zn–Se interlayer was present (as deduced by the In/As elemental ratio, which increased from 1.07 to 1.83 during the ZnSe shelling process, Table S1). These QDs had a PLQY as high as 60% and single-exponential decay kinetics with a 52 ns lifetime (Figure 3b,c and Table 3) comparable to the In­(Zn)­As@ZnSe QDs shown in Figure 2. Also, in this case, a broadening of the excitonic absorption peak was observed upon ZnSe shelling (Figure S6).

**Figure 3:**
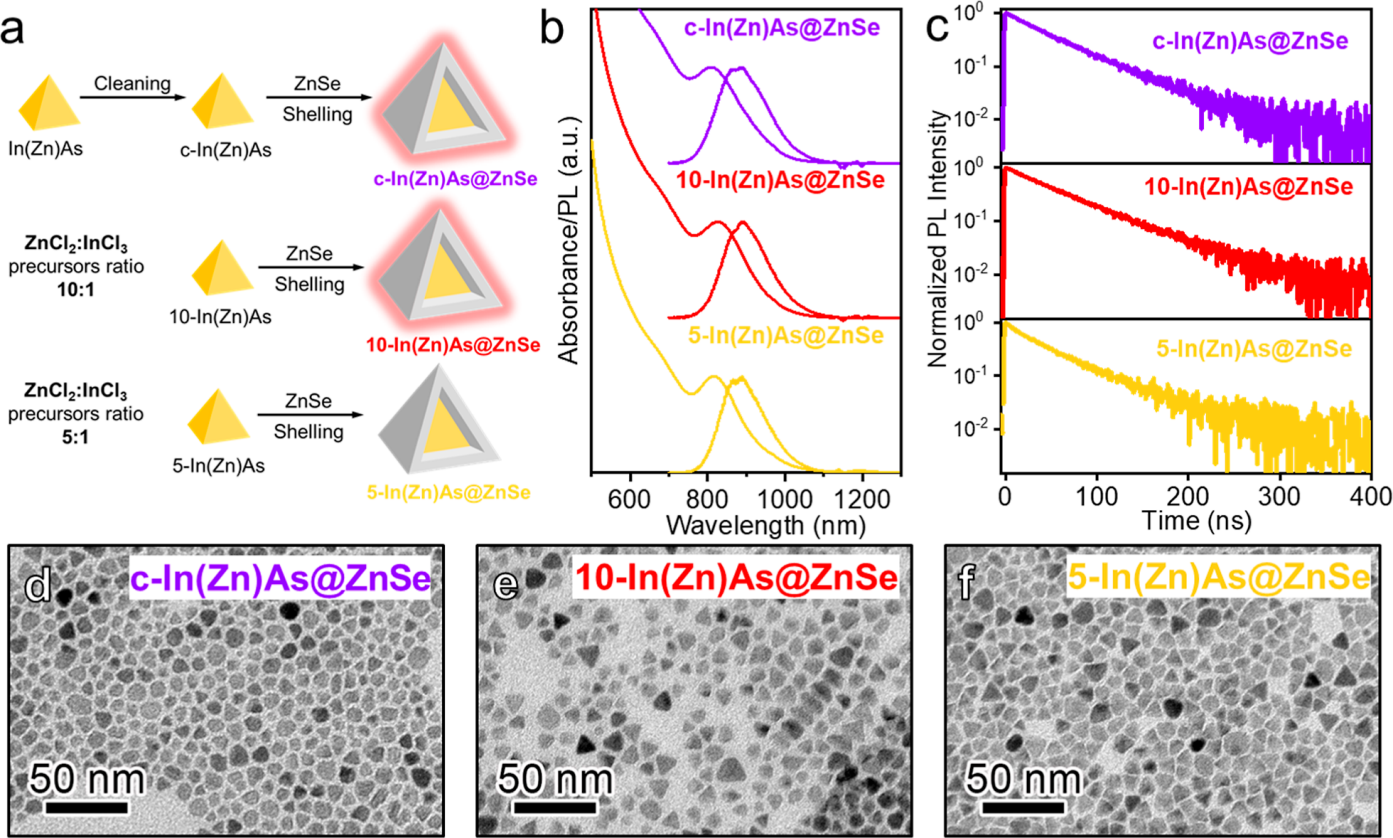
(a) Schematic representation of the synthesis process to prepare c-In­(Zn)­As@ZnSe, 10-In­(Zn)­As@ZnSe, and 5-In­(Zn)­As@ZnSe QDs. Optical characterization of c-In­(Zn)­As@ZnSe, 10-In­(Zn)­As@ZnSe, and 5-In­(Zn)­As@ZnSe QDs; (b) Optical density and PL spectra; (c) PL decay traces. TEM images of (d) c-In­(Zn)­As@ZnSe, (e) 10-In­(Zn)­As@ZnSe, and (f) 5-In­(Zn)­As@ZnSe QDs.

**Table 3:** Atomic Ratios, Size, ZnSe Thickness, and Optical Data of c-In­(Zn)­As@ZnSe, 10-In­(Zn)­As@ZnSe, and 5-In­(Zn)­As@ZnSe QD Samples

sample	In/As ratio^a^	Zn/As ratio^a^	Zn/Se ratio^a^	size (nm)	ZnSe ML	PLQY (%)	PL lifetime at RT
c-In(Zn)As@ZnSe	1.83	24.49	1.07	9.2 ± 1.4	7	60 ± 6	τ = 57 ns
10-In(Zn)As@ZnSe	2.08	21.97	1.05	9.2 ± 1.3	6.5	70 ± 7	τ = 52 ns
5-In(Zn)As@ZnSe	2.34	26.32	1.00	8.8 ± 1.5	7	22 ± 5	τ_1_ = 9 ns (4%), τ_2_ = 52 ns (96%)

a The atomic ratios were measured via ICP-OES analysis.

These findings suggest that In­(Zn)As QDs themselves, rather than the specific shelling process employed, possess the intrinsic capability to reach high PLQY values upon ZnSe overgrowth. Moreover, our data indicate that the formation of an In–Zn–Se interlayer and the continuous increase of the In/As ratio during ZnSe shell growth, observed in all the samples discussed so far, imply that In must be sourced from the starting QDs, for example, through etching. This process could be related to the broadening of the excitonic absorption peaks observed during ZnSe shelling. Indeed, a similar etching phenomenon was very recently reported by Li et al., who observed broadening of the excitonic absorption peak when growing ZnSe shells onto InAs QDs prepared with TMS-As.^34^ Notably, the broadening of the excitonic peak was less pronounced in In­(Zn)As and c-In­(Zn)­As QDs compared to that in std-InAs and Zn–InAs QDs (Figure S6). This suggests that etching may proceed differently depending on the “nature” of the starting InAs cores, although the precise mechanism remains unclear and will require further investigation.

In the second set of experiments, we synthesized In­(Zn)As QDs using lower amounts of ZnCl_2_ compared to previous experiments, where a ZnCl_2_:InCl_3_ precursor ratio of 20:1 was used. Specifically, we tested ZnCl_2_:InCl_3_ precursor ratios of 10:1 (sample 10-In­(Zn)­As) and 5:1 (sample 5-In­(Zn)­As) (Figure 3a lower schemes; see also the Materials and Methods section for details). Both QD samples were overcoated with a ZnSe shell using our “one-pot” shelling procedure, resulting in core@shell QDs with a shell thickness of 7 ML and an In–Zn–Se “interlayer” (i.e., In/As elemental ratios of 2.08 and 2.34, see Table 3 and Figures 3d–f and S7). However, their PLQY values differed significantly: 10-In­(Zn)­As@ZnSe reached a PLQY as high as 70 ± 7% with a single-exponential PL decay with a PL lifetime of 57 ns, while 5-In­(Zn)­As@ZnSe had a lower PLQY of 22 ± 5% with a double exponential PL decay with a fast, 4 ns, decay followed by a 52 ns component similar to the high PLQY counterparts, which is indicative of residual carrier trapping due to insufficient surface passivation (Figure 3b,c and Table 2). These control experiments confirm the essential role of ZnCl_2_ as an additive in the synthesis of InAs QDs. To achieve high PL efficiency, a Zn:In precursor ratio of at least 10:1 is required, as this facilitates effective passivation of surface defects, as indicated by TRPL measurements (Figure 3c and Table S2). Moreover, when InAs QDs are prepared with ZnCl_2_, the specific procedure adopted to grow the ZnSe shell plays only a minor role, with the “one-pot” method yielding slightly more emissive QDs compared with the “two-pot” method.

### Simulation of InAs Core QDs

To shed light on the role of Zn location in the optical properties of InAs QDs, we performed density functional theory (DFT/HLE17) calculations. Based on the shapes observed in the TEM images reported in Figure S2 and in our previous work,^31^ we initially modeled a truncated tetrahedral InAs QD of ∼4 nm height exhibiting (100), (111), and (−1–1–1) facets passivated with Cl^–^ ions and neutral methylamine (MA) molecules, the latter mimicking the oleylamine used in the synthesis. To ensure that the initial InAs QD model had a In/As ratio of approximately 1.15, similar to the experimentally measured value (Tables 1, S2, and S3), we removed InCl_3_ from the In-rich (100) facets, in line with our previous works (Figure 4a).^30–33^ In this model, the As-rich (−1–1–1) facets are characterized by a significant accumulation of net negative charge due to the dangling bonds of the surface As ions and consequently by the presence of facet-specific trap states (Figure 4a–c). Indeed, as demonstrated by Llusar et al.,^35^ this charge accumulation energetically shifts the molecular orbitals localized on these atoms, causing them to decouple from core orbitals and to appear in the bandgap as facet-specific trap states, on top of the valence band (Figure 4b,c).^31,32^ This model is consistent with the negligible PL and the presence of surface traps characterizing std-InAs QDs.

**Figure 4:**
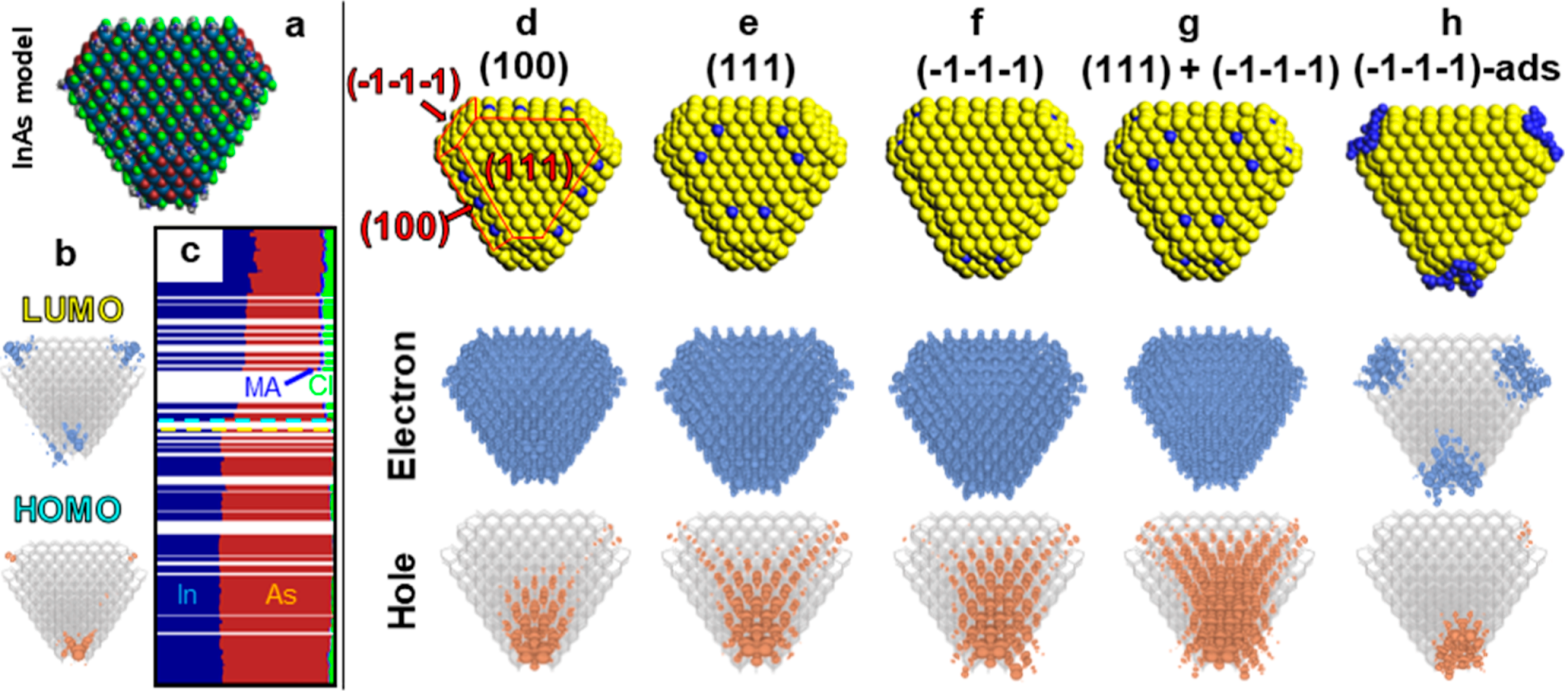
(a) InAs model (In_780_As_691_Cl_267_MA_105_) with corresponding (b) LUMO and HOMO charge densities and (c) projected density of states plot. Horizontal bars indicate MO contributions by element: In (dark blue), As (brown), MA (N: blue, C: gray, H: white), Cl (green). The Fermi level is shown as a dashed line. Effect on carriers’ distribution of surface Zn incorporation at (d) (100), (e) (111), (f) (−1–1–1), or (g) (111) + (−1–1–1) facets and (h) of ZnCl_2_ adsorbed onto (−1–1–1) facets. Top row: model configurations (yellow = InAs, blue = Zn). Note that Cl^–^ ions and MA ligands are omitted for clarity in (d–g). Middle and bottom rows: electron and hole charge densities.

To analyze the role of Zn incorporation in the InAs structure, we first considered whether Zn atoms are more readily incorporated into the lattice of InAs QDs or tend to remain on the surface. To this aim, we built a simple InAs QD model (Figure S8) and substituted an In atom in various locations of the QD with a Zn atom. Our results indicated that Zn is unlikely to occupy lattice positions inside the QD. Indeed, the migration of a Zn atom from the surface to the inner layer of the QD brings about an energy penalty of at least 5.16 kcal/mol·atom (Table S2). These findings are also consistent with those of the X-ray absorption near edge structure (XANES) and extended X-ray absorption fine structure (EXAFS) experiments (vide infra).

We therefore focused our investigation on the effects of placing Zn atoms on the surface of the InAs QD model reported in Figure 4a. In practice, on the (100) facet, this involved filling InCl_3_ vacancies with ZnCl_2_ moieties; on the (−1–1–1) facets, ZnCl_2_ would replace the position occupied by an InAs pair; on the (111) facet, this consisted of substituting surface In atoms with Zn atoms. In this process, the In/As and Zn/As ratios were kept fixed at ∼1.11 and 0.08, respectively, in line with the experimental values (Table S3). We explored different intermediate InAs QD models where Zn could be incorporated: (i) on either the (100), (111), or (−1–1–1) facets separately; (ii) in mixed configurations involving (100) + (111), (100) + (−1–1–1), or (111) + (−1–1–1) facets (Figures 4d–g and S9 upper rows). Across all configurations, the electron charge density (LUMO) remained largely unaffected and uniformly delocalized (Figures 4d–g and S9 middle rows). However, hole delocalization (HOMO) varied depending on the Zn incorporation sites: incorporation at the (111) and (−1–1–1) facets resulted in improved wave function delocalization, which was maximized when Zn atoms were simultaneously placed on both facets (Figures 4g and S9 bottom rows).

To clarify this improvement, particularly at the (−1–1–1) facets, which were responsible for most facet-specific trap states in the reference InAs QD model (Figure 4a,b), we analyzed how negative charge accumulated on this facet before and after Zn treatment. By replacing one surface As ion and the underlying In ion (i.e., a neutral InAs pair) with a neutral ZnCl_2_ unit and thus incorporating Zn in the lattice, the negative charge of the (−1–1–1) facets is reduced. Focusing specifically on the dangling atoms, substituting one As^3–^ with two Cl^–^ ions reduces the negative charge from −3 to −2 per InAs unit being replaced. This overall reduction in net negative charge, further enhanced by the replacement of several InAs units with ZnCl_2_ per facet, promotes hole delocalization by enabling the surface orbitals of the (−1–1–1)-facets to mix with the core orbitals, effectively suppressing the facet-specific trap (Figure 4g).

This charge reduction is achieved when ZnCl_2_ species effectively replace lattice sites at the surface. We also considered the effect of ZnCl_2_ species simply adsorbed on the (−1–1–1) facet, a process that does not involve replacing InAs pairs with ZnCl_2_ (Figure 4h), where ZnCl_2_ acts as a Z-type ligand. Overall, our DFT results indicated that simple adsorption of ZnCl_2_ does not enhance charge delocalization as it does not affect the accumulation of charges on this facet.

Based on these results, we hypothesize the following: (i) the direct use of ZnCl_2_ during the synthesis of InAs QDs likely leads to ZnCl_2_ incorporation on all facets, including (−1–1–1) and (111), since ZnCl_2_ is present throughout the QDs growth. This likely explains the appreciable PL emission and surface traps passivation exhibited by In­(Zn)As QDs; (ii) the use of ZnCl_2_ in the postsynthesis treatment of std-InAs QDs (the latter representing our reference InAs QD model in the calculations), instead, likely results in only minor surface Zn incorporation, primarily at surface areas with greater extension, such as the (100) facets, while on the As-rich (−1–1–1) facets, ZnCl_2_ is simply adsorbed. This insight might explain why std-InAs and Zn–InAs QDs retain charges on the (−1–1–1) facets, leading to facet-specific traps and consequently very weak PL.

### Structure Information on InAs Core QDs

To experimentally investigate the Zn location in our InAs QDs, XAS experiments on Zn K-edge were conducted on In­(Zn)As and Zn–InAs QD samples. Both XANES and EXAFS spectra were collected across the Zn K-edge at 9.6586 keV. XANES spectra of both QD samples exhibited three distinct peaks, labeled A, B, and C, located at 9665.0(5), 9669.9(5), and 9680.9(5) eV, respectively (Figure 5a and Supporting Information for details).

To interpret the minor differences between the spectral shapes of the two samples, we performed XANES calculations using structures derived from DFT calculations (see previous section), considering the Zn incorporation at specific facets, namely, (100), (111), and (−1–1–1) as well as the ZnCl_2_ adsorption onto the (−1–1–1) facets, without accounting for experimental broadening (Figure 5a). It is worth anticipating here that XANES and EXAFS results, discussed below, excluded the incorporation of Zn atoms in the lattice of InAs QDs, in agreement with DFT calculations that found this to be energetically unfavorable (see previous section). Features A and B, with varying amplitudes and slight shifts in position, were present in all the calculated spectra where Zn atoms were incorporated at the different facets but were absent in the spectrum where ZnCl_2_ was adsorbed onto the (−1–1–1) facets (Figure 5a). The feature C was clearly identified only when incorporating Zn at the (111) and (100) facets (Figure 5a). Given that all the experimental spectra displayed features A, B, and C, albeit with feature C slightly shifted to lower energies compared to the simulated spectra, we concluded that Zn atoms in In­(Zn)­As and Zn–InAs QD samples were likely distributed across all three facets, with slightly different proportions in each sample. In order to assess the Zn occupation on the different facets of the two QD samples, we fitted the EXAFS data, using the simplified model described in the Experimental Section (see also Table S4). The results are shown in Figure 5b,c, and the fitted structural parameters are given in Table 4. It is worth specifying that the Debye–Waller factor is related to disorder (in terms of bond distances), with lower values indicating lower disorder. The amplitude is directly proportional to the coordination number of a given atom (Zn in this case) (see footnote a of Table 4).

**Table 4:** Calculated and Experimental Crystallographic Parameters Derived from the Zn K-Edge EXAFS Fitting of the In­(Zn)­As and Zn–InAs Samples

sample	path	amplitude^a^ ^,^ ^b^	distance (Å)^b^	Debye–Waller factor (Å^2^)^b^
In(Zn)As	Zn–Cl	2.4(2)	2.143(7)	0.0121(8)
	Zn–As	1.14(7)	2.418(3)	0.0042(3)
Zn–InAs	Zn–Cl	2.6(2)	2.139(5)	0.0117(7)
	Zn–As	0.96(6)	2.419(3)	0.0037(3)

a The amplitude is the product of the scaling factor S02 and the coordination number (cf. Table S3).

b The fit correlation factor *R* in *R*-space is equal to 0.018 over the *k* range of [3.538; 13.142] Å^–1^ and *R* range of [1.25; 2.581] Å.

**Figure 5:**
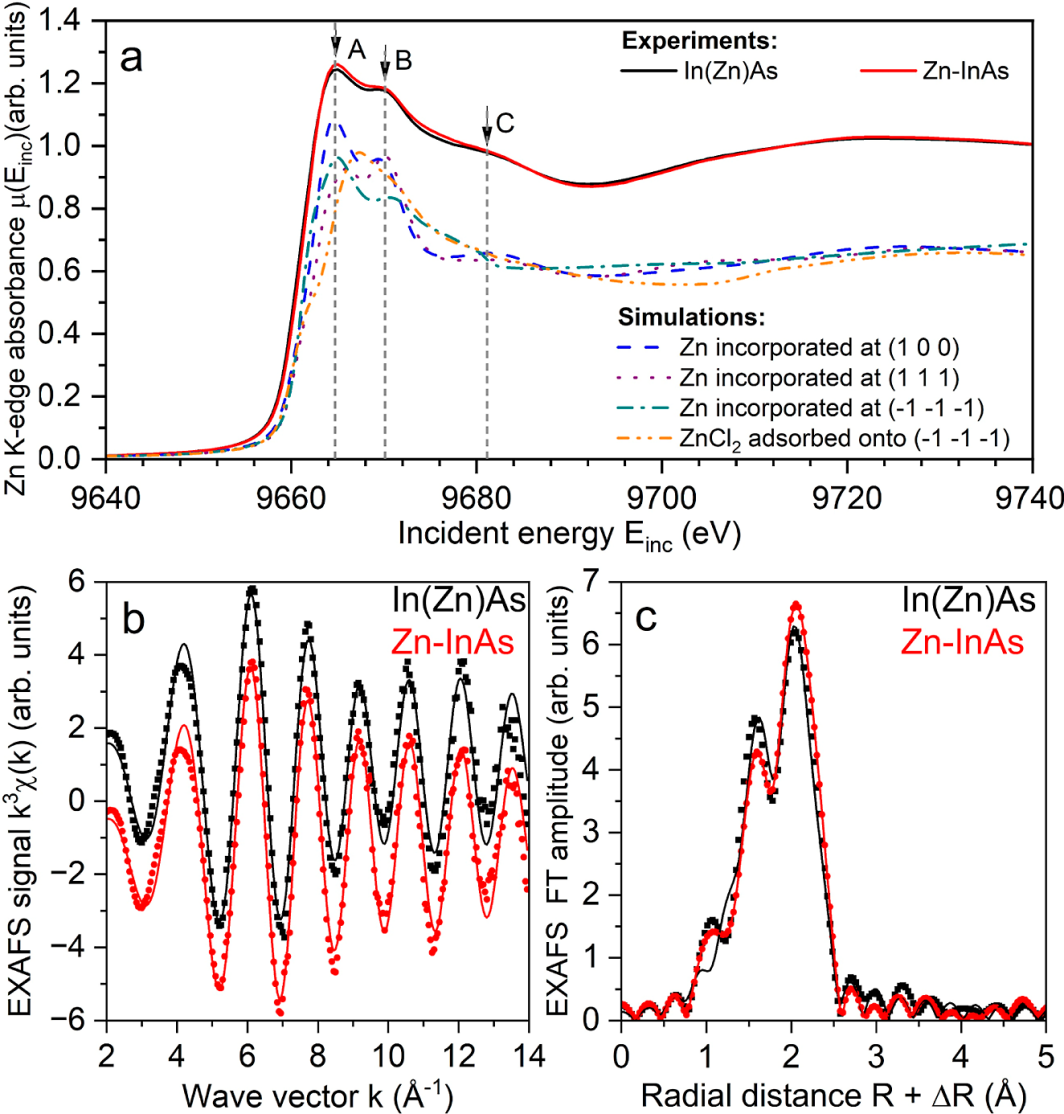
(a) Experimental Zn K-edge XANES spectra (black and red curves) and calculated Zn K-edge XANES spectra for Zn incorporation at the (100), (111), or (−1–1–1) facets and for ZnCl_2_ adsorption onto the (−1–1–1) facets. (b) Zn K-edge *k* ^3^-weighted EXAFS data and (c) the corresponding Fourier transform (FT). Experimental data are shown as dots, while best-fit results are represented by lines. The distances are not phase-corrected. The FT window and fitting range were [3.5; 13] Å^–1^ and [1; 2.6] Å, respectively.

The short Zn–As distance and its small Debye–Waller factor, showing limited structural disorder, resulting from the EXAFS fitting of the two samples indicated that (i) Zn was not incorporated in the lattice (i.e., core) of these InAs QD samples as this would have resulted in Zn–As distances of at least 2.7 Å (without considering possible distortions); (ii) in both samples, Zn atoms were primarily incorporated at the (100) and (−1–1–1) facets and not at the (111) facets, as the latter would have entailed longer Zn–As distances. As expected from the comparison of the XANES spectra, the proportion of atoms occupying each facet differs depending on the QD preparation. A comparison of the different fitted amplitudes shows a significant decrease of the Zn–As path for Zn–InAs compared to In­(Zn)As QDs (Table 4). This can be explained considering that a decrease of the second shell coordination number occurs not only when Zn is preferentially incorporated at the (−1–1–1) facets relative to the (100) facets (coordination numbers being about 3 and 2, respectively) but also when ZnCl_2_ is adsorbed onto the (−1–1–1) facets instead of being fully incorporated (coordination numbers being about 2 and 1, respectively). In addition, the Zn–Cl amplitude was slightly higher in Zn–InAs compared to In­(Zn)­As, which could be explained considering Zn to be present in the form of ZnCl_2_ adsorbed onto the (−1–1–1) facets, rather than being incorporated at the (−1–1–1) facets. Therefore, the EXAFS results suggested that Zn incorporation at the (−1–1–1) and (100) facets occurs to a greater extent in In­(Zn)As QDs compared to Zn–InAs QDs, where ZnCl_2_ adsorption onto the (−1–1–1) facets is most likely to take place, in agreement also with the XPS results (Figure 1c).

Based on the XAS results, combined with DFT insights as well as structural and optical data, we can conclude the following:

- The use of ZnCl_2_ during InAs QD synthesis leads to Zn being incorporated at the surface of InAs QDs with a preference for the (100) and (−1–1–1) facets. This results in In­(Zn)As QDs with efficient surface traps passivation, which are capable, upon ZnSe shelling, of achieving high PLQY.
- Postsynthesis treatment of std-InAs QDs with ZnCl_2_ results in limited surface Zn incorporation (less than that observed for In­(Zn)As QDs), with a preference for the (100) and (−1–1–1) facets, along with concurrent ZnCl_2_ adsorption onto the (−1–1–1) facets. As a consequence, Zn–InAs QDs, like std-InAs QDs, exhibit inefficient surface traps passivation, which is partially retained upon ZnSe shell growth, ultimately leading to InAs@ZnSe core@shell QDs with low PLQYs.

## Conclusion

In this work, we investigated how ZnCl_2_, employed as an additive in the amino-As-based synthesis of InAs QDs, is capable of boosting the PL efficiency of the resulting InAs@ZnSe core@shell QDs. This was done by synthesizing and comparing the optical and structural properties of three different types of InAs QDs and the corresponding InAs@ZnSe core@shell structures: (i) InAs QDs produced with ZnCl_2_ as an additive (In­(Zn)­As); (ii) InAs QDs synthesized without additives (std-InAs); (iii) InAs QDs synthesized with no additive and subsequently postsynthesis treated with ZnCl_2_ (Zn–InAs). Our work indicates that high PLQY values could be attained only in the case of In­(Zn)­As@ZnSe QDs (∼70%), while std-InAs@ZnSe and Zn–InAs@ZnSe samples reached low PL efficiencies (max 20%). Interestingly, contrary to what was hypothesized in our previous work,^30^ the high PLQY characterizing In­(Zn)­As@ZnSe QDs could not be ascribed solely to the presence of an In–Zn–Se interlayer as this was found also in std-InAs@ZnSe and Zn–InAs@ZnSe QDs. Our findings also indicate that performing the ZnSe shelling via a two-pot rather than a one-pot procedure has a minor effect on the PLQY of the final In­(Zn)­As@ZnSe QDs. Notably, their PL efficiency could only be significantly increased when high amounts of ZnCl_2_ additive were used during their synthesis (specifically, when the ZnCl_2_:InCl_3_ precursor ratio was higher than 10:1).

These results were rationalized via DFT calculations coupled with X-ray absorption measurements which revealed that (i) std-InAs QDs feature surface trap states, mainly located at the (−1–1–1) facets, accounting for their low PL efficiency, even after ZnSe shelling; (ii) the postsynthesis treatment of std-InAs QDs with ZnCl_2_ results in limited surface Zn incorporation and in ZnCl_2_ adsorption onto the (−1–1–1) facets, leading to a poor surface trap passivation and thus to poorly emissive Zn–InAs@ZnSe QDs; (iii) employing ZnCl_2_ as an additive in the synthesis of InAs QDs leads to the incorporation of Zn atoms at the surface of In­(Zn)As QDs, with a preference for the (100) and (−1–1–1) facets, which results in effective surface traps passivation, and, consequently, to strongly emissive In­(Zn)­As@ZnSe QD systems.

Overall, our study demonstrates the critical role of ZnCl_2_ as an additive in the synthesis of amino-As-based InAs QDs, enabling the preparation of strongly emissive InAs@ZnSe QDs.

## Materials and Methods

### Chemicals

Indium­(III) chloride (InCl_3_, 99.999%, Sigma-Aldrich), zinc­(II) chloride (ZnCl_2_, 99.999%, Sigma-Aldrich), tris­(dimethylamino)­arsine (amino-As, 99%, Strem), alane *N*,*N*-dimethylethylamine complex solution (DMEA-AlH_3_, 0.5 M solution in toluene, Sigma-Aldrich), selenium powder (Se, 99.99%, Strem), triethyloxonium tetrafluoroborate (Et_3_OBF_4_, 97%, Sigma-Aldrich), oleylamine (OA, 98%, Sigma-Aldrich), tri-*n*-octylphosphine (TOP, 97%, Strem), toluene (anhydrous, 99.8%, Sigma-Aldrich), ethanol (anhydrous, 99.8%, Sigma-Aldrich), hexane (anhydrous, 95%, Sigma-Aldrich), and *N*,*N*-dimethylformamide (DMF, anhydrous, 99.8%, Sigma-Aldrich). All of the chemicals were used without further purification.

### Preparation of the 0.4 M As Precursor

In a N_2_-filled glovebox, 0.2 mmol of amino-As (37 μL) was dissolved in 0.5 mL of degassed oleylamine at 40 °C for 5 min until no bubbles further evolved.

### Preparation of the 1 M TOP–Se Precursor

In a N_2_-filled glovebox, 10 mmol of Se powder was mixed with 10 mL of TOP in a 20 mL glass vial and heated at 250 °C under constant stirring for ≈30 min to form a transparent solution, and then the mixture was cooled to room temperature.

### Preparation of the 0.8 M ZnCl_2_–OA Precursor

In a N_2_-filled glovebox, 8 mmol of ZnCl_2_ was mixed with 10 mL of OA in a 20 mL glass vial and heated at 250 °C under constant stirring for ≈50 min. Because the 0.8 M ZnCl_2_–OA precursor solidified at room temperature, it was preheated before being transferred into a syringe.

### Synthesis of InAs QDs

Std-InAs QDs and In­(Zn)As QDs were synthesized following the procedure reported in a former work of our group.^30^ In a typical synthesis, 0.2 mmol of InCl_3_, *X* mmol of ZnCl_2_ (*X* = 0 for std-InAs and 4 for In­(Zn)­As), and 5 mL of OA were loaded into a 100 mL three-necked flask under an inert atmosphere. The mixture was degassed at room temperature for 10 min and then at 120 °C under vacuum for 40 min. Next, the flask was heated up to 180 °C under N_2_ to completely dissolve all the precursors, and then it was cooled to 120 °C and dried under vacuum for extra 30 min. The mixture was heated to 240 °C under nitrogen, and the As precursor was injected into the flask, quickly followed by the injection of 1.2 mL of a DMEA-AlH_3_ toluene solution. The temperature was quickly increased to 300 °C (≈30 °C min^–1^), the reaction was then allowed to run for 15 min, and it was quenched by removing the heating mantle. For Zn–InAs QDs, 5 mL of 0.8 M ZnCl_2_–OA precursor was injected into std-InAs QDs crude solution at around 90 °C. The solution was heated up to 280 °C (≈30 °C min^–1^) and kept at 280 °C for 3 h, after which the reaction was quenched by removing the heating mantle. The QDs were washed twice by the addition of toluene and ethanol and precipitated by centrifugation at 4000 rpm. The final product was dispersed in toluene for further characterization.

### Synthesis of InAs@ZnSe Core@Shell QDs

After quenching the growth of the InAs QDs by cooling the reaction mixture to 90 °C, 3.5 mL of 0.8 M ZnCl_2_–OA was injected into the flask followed by the injection of 7.5 mL of TOP–Se. The mixture was heated to 310 °C (≈30 °C min^–1^) and kept at 310 °C for 2 h. The InAs@ZnSe core@shell QDs were washed by the addition of toluene and ethanol and precipitated by centrifugation at 2000 rpm two times. The final product was dispersed in toluene for further characterization.

### Ligand-Stripping Procedure

In a N_2_-filled glovebox, 0.5 mL of a Zn–InAs QDs dispersion (in toluene) was added to 1 mL of hexane in a glass vial, and then 1 mL of a solution of Et_3_OBF_4_ in DMF (100 mm) was added into the vial. After the vial was shaken for several seconds, the QDs were transferred from the hexane into the DMF phase. The QDs dispersed in DMF were precipitated by the addition of toluene, followed by centrifugation at 4000 rpm for 5 min. To remove residual organic ligands, the washing procedure was repeated twice, and the resulting QDs were dispersed in DMF.

### Powder XRD

XRD patterns were acquired with a PANanalytical Empyrean X-ray diffractometer equipped with a 1.8 kW Cu Kα ceramic X-ray tube and a PIXcel3D 2 × 2 area detector, operating at 45 kV and 40 mA. Specimens for XRD measurements were prepared by dropping a concentrated QDs solution onto a silicon zero-diffraction single-crystal substrate. The diffraction patterns were recorded under ambient conditions using a parallel beam geometry and symmetric reflection mode. XRD data analysis was performed using the HighScore 4.1 software from PANalytical.

### Transmission Electron Microscopy Characterization

Diluted QDs dispersions were drop-cast onto copper TEM grids with an ultrathin carbon film. Low-resolution TEM images were acquired on a JEOL JEM-1400Plus microscope with a thermionic gun (W filament) operated at an acceleration voltage of 120 kV.

### Inductively Coupled Plasma Optical Emission Spectroscopy (ICP-OES)

The elemental analysis was carried out via inductively coupled plasma optical emission spectroscopy (ICP-OES) with an iCAP 7600 DUO ICPOES spectrometer (Thermo Fisher Scientific). The samples were dissolved in 1 mL of HNO_3_ overnight and then diluted with 9 mL of Milli-Q water for measurements. The elemental analysis using ICP-OES was affected by a systematic error of ≈5%.

### X-ray Photoelectron Spectroscopy

XPS analysis was performed on a Kratos Axis UltraDLD spectrometer using a monochromatic Al Kα source (20 mA and 15 kV). Survey scan analyses were carried out over an analysis area of 300 × 700 μm and a pass energy of 160 eV, whereas high-resolution analyses were conducted with a pass energy of 10 eV. Specimens for XPS were prepared from concentrated NC solutions, dropped on freshly cleaved highly oriented pyrolytic graphite substrates in a glovebox. The Kratos charge neutralizer system was used on all specimens. Spectra were charge-corrected to the main line of the carbon 1s spectrum (adventitious carbon) set to 284.8 eV. Spectra were analyzed using CasaXPS software (version 2.3.24).

### Optical Characterization

The absorption spectra were recorded on a Varian Cary 5000 UV–vis–NIR spectrophotometer. The samples were prepared by diluting NC samples in 3 mL of toluene in 1 cm path length quartz cuvettes with airtight screw caps in a N_2_-filled glovebox. The steady-state measurements were carried out on an Edinburgh Instruments FLS900 fluorescence spectrometer equipped with a Xe lamp. Absolute PLQY measurements were performed using the Edinburgh FLS920 fluorescence spectrometer equipped with an integrating sphere and a PMT-1700 liquid nitrogen-cooled detector. The samples were excited at 700 nm using the output of continuous xenon lamp. Both the scattering (excitation) peak at 700 nm and the PL of each sample were measured relative to those of a blank standard (a cuvette containing only the solvent), using the same PMT-1700 detector, in order to determine the absolute PLQY. All QDs solutions were diluted to an optical density of ≈0.1 at 700 nm. Time-resolved μ-PL spectroscopy of QD samples: A pulsed laser PiL040-FS with an emission wavelength centered at 510 nm and a 1 MHz repetition rate with a pulse width of <45 ps from NKT Photonics was used for excitation. The PL emission was collected, and an avalanche photodiode coupled to the PicoHarp 300 (PicoQuant) time-correlated single photon counting system was used for detection.

### X-ray Absorption Spectroscopy

XAS analyses were performed at the BM20 (The Rossendorf Beamline) of the European Synchrotron Radiation Facility (ESRF) operating at an electron beam energy of 6 GeV, in Grenoble, France. The incident energy was scanned using a Si(111) monochromator.^36^ Experiments were performed at room temperature in the so-called fluorescence mode by detecting the Zn KL3 emission line at 8.6389 keV using the multielement high-purity germanium fluorescence detector available at BM20. Energy calibration was performed using the first inflection point of the energy derivative of the K-edge excitation energy of Zn metallic foil at 9658.6 eV. The detected intensity was normalized to the incident photon flux.

The XANES theoretical calculations were performed using the finite difference method for the near-edge structure code.^37^ An atomic cluster of 7 Å was used in self-consistent-field calculations using the Dirac–Slater approach. The Poisson equation was solved to obtain the Coulomb potential from the superposed self-consistent atomic densities in the considered cluster. The energy-dependent exchange–correlation potential was evaluated by using the local density approximation and constructed using both the real Hedin–Lundquist and Von Barth formulations. These calculations were based on static atom supercells of hundreds of atoms derived from the DFT calculations, i.e., the optimized structure with 50 Zn atoms occupying all facets, and thermally induced disorder was not considered. Because of the presence of heavy nuclei (In), spin–orbit effects were considered, but no spin-polarization effect has been noticed. The calculated spectra were finally convoluted by the core-hole lifetime and the continuum “arctangent” model.

### Density Functional Theory Calculations

DFT calculations were performed using the meta-GGA high-local-exchange 2017 functional (HLE17).^38^ A double-ζ basis set (DZVP) and the Gaussian and plane waves method (GPW) were employed, as implemented in the CP2K 2024.1 quantum chemistry package.^39^ Relativistic effects were included via the effective core potentials. Geometry optimizations were carried out in the gas phase using cubic simulation boxes that extended at least 10 Å beyond the outermost atoms of the InAs QD models. Structures were relaxed until the following convergence criteria were met: maximum force of 4.5 × 10^–4^ Ha/bohr, root-mean-square (rms) force of 3.0 × 10^–4^ Ha/bohr, maximum step size of 3.0 × 10^–3^ bohr, and rms step size of 1.5 × 10^–3^ bohr. The isosurface value used for the charge density plots was |0.005| (e^–^/bohr^3^)^1/2^.

## Supplementary Material



## Notes

XRD patterns and TEM micrographs of core InAs QDs, postsynthesis treatment of std-InAs with ZnCl_2_, XRD patterns of c-In­(Zn)­As@ZnSe, 10-In­(Zn)­As@ZnSe, and 5-In­(Zn)­As@ZnSe QDs, XPS spectra of ZnCl_2_ and oxidized Zn–InAs, absorption curves and elemental analyses of samples during ZnSe shelling, atomistic models used for DFT, carriers distribution upon placing Zn atoms on InAs models, and XANES and EXAFS curves fitting (PDF)
