# Supplementary material for: Unveiling the Role of ZnCl2 in Enhancing the Photoluminescence Efficiency of Amino-As-Based InAs@ZnSe Quantum Dots
Source: ACS Nano. 2025 Sep 25;19(39):34807–18. doi: 10.1021/acsnano.5c10371 (PMC12509302; doi:10.1021/acsnano.5c10371)
Supplement: Supplementary file 1 [file nn5c10371_si_001.pdf]

Supporting Information

Unveiling the Role of $ZnCl_2$ in Enhancing the Photoluminescence Efficiency of Amino-As-Based InAs@ZnSe Quantum Dots

Dongxu Zhu[a‡], Jordi Llusar[b‡], Aswin Asaithambi[a], Zheming Liu[a], René Bes[c], Damien Prieur[d,e], Hiba H. Karakkal[f], Mirko Prato[g], Sergio Brovelli[f], Gabriele Saleh[a], Satyaprakash Panda[a,h], Ivan Infante*[b,i], Luca De Trizio*[j], Liberato Manna*[a]

[a] Nanochemistry,[g] Materials Characterization and [j] Chemistry Facility, Istituto Italiano di Tecnologia, Via Morego 30, 16163 Genova, Italy

[c] Department of Physics and Helsinki Institute of Physics, University of Helsinki, P.O. Box 43, FI-00014 Helsinki, Finland

[d] HZDR, Institute of Resource Ecology, Bautzner Landstraße 400, 01328 Dresden, Germany

[e] Rossendorf beamline (BM20-CRG), European Synchrotron Radiation Facility, avenue des Martyrs 71, 38043 Grenoble, France

[f] Dipartimento di Scienza dei Materiali, Università degli Studi di Milano-Bicocca, Via R. Cozzi 55, 20125, Milano, Italy

[b] BCMaterials, Basque Center for Materials, Applications, and Nanostructures, UPV/EHU Science Park, Leioa 48940, Spain

[h] Dipartimento di Chimica e Chimica Industriale, Università di Genova, 16146 Genova, Italy

[i] Ikerbasque Basque Foundation for Science Bilbao 48009, Spain

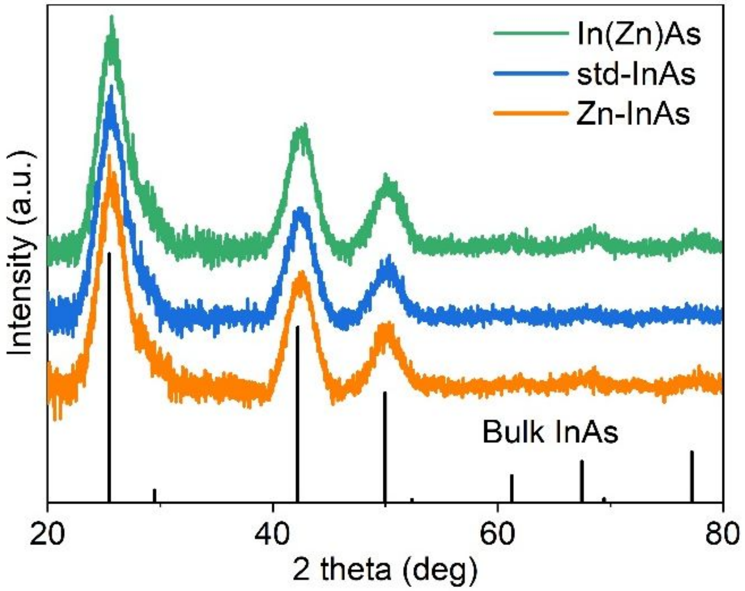

Figure S1. XRD pattern of In(Zn)As QDs, std-InAs QDs and Zn-InAs QDs with the bulk reflections of InAs (ICSD 98-002-4518).

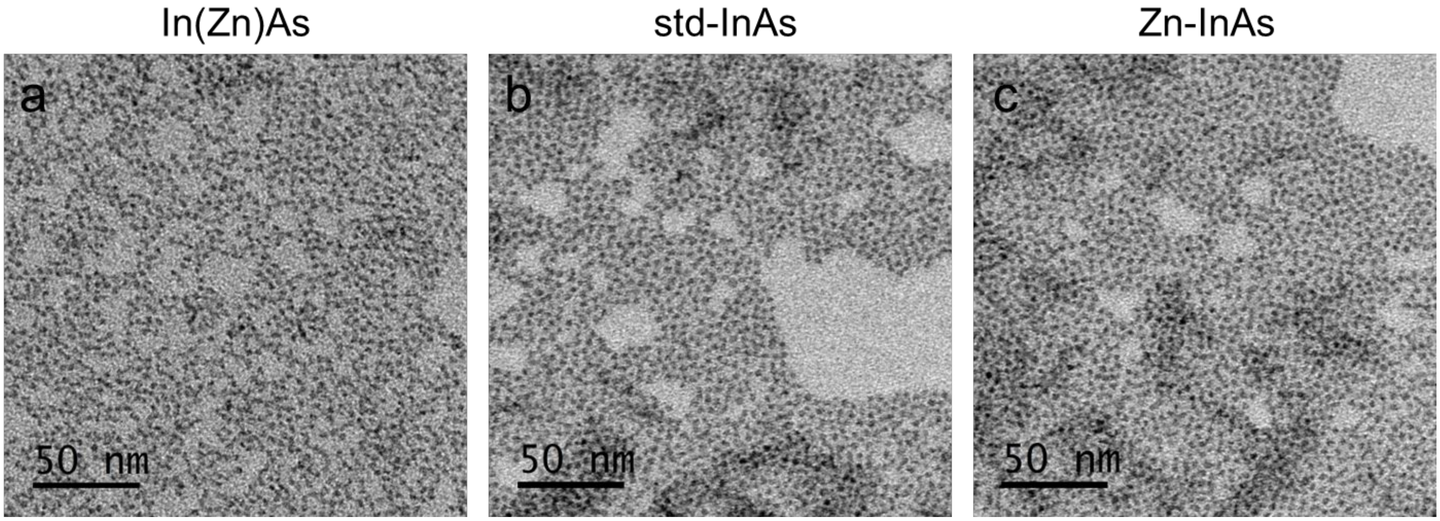

Figure S2. TEM images of a) In(Zn)As QDs, b) std-InAs QDs and c) Zn-InAs QDs.

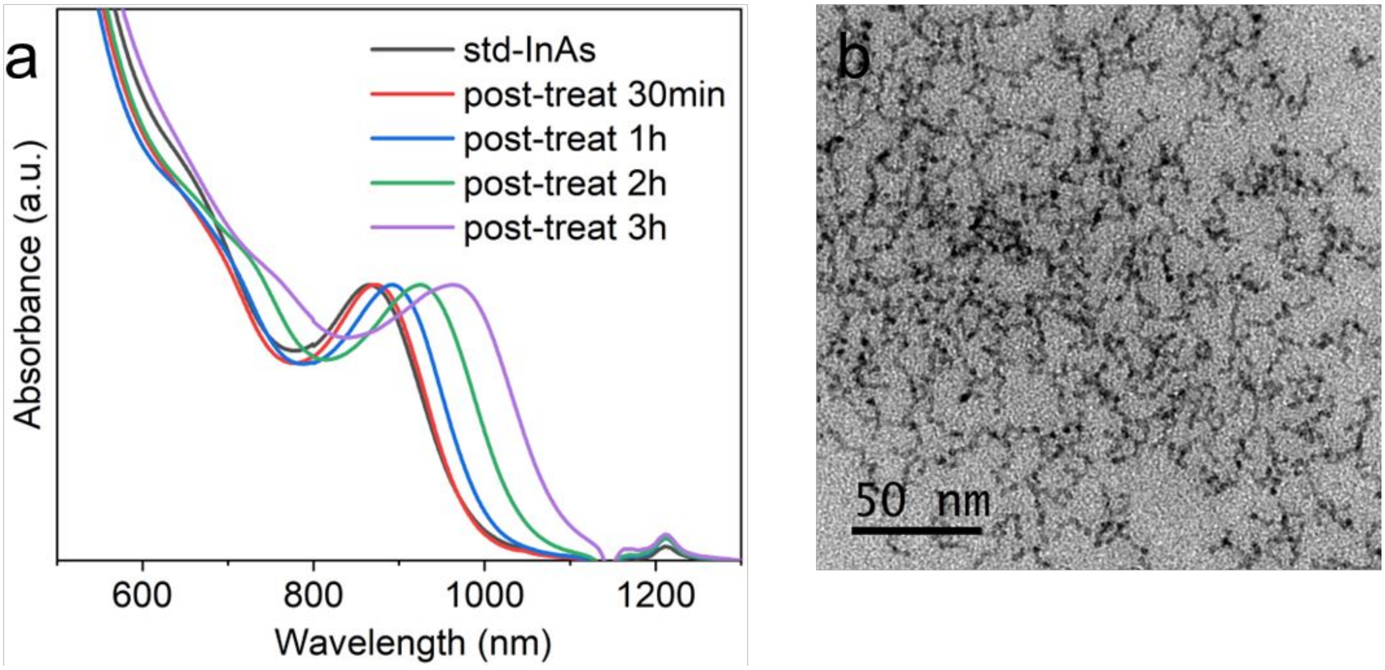

Figure S3. a) Absorption curves of std-InAs QDs subjected to $ZnCl_2$ post synthesis treatment at 300°C for different time. b) TEM image of the resulting InAs QDs after a post-synthesis treatment at 300°C for 3 hours.

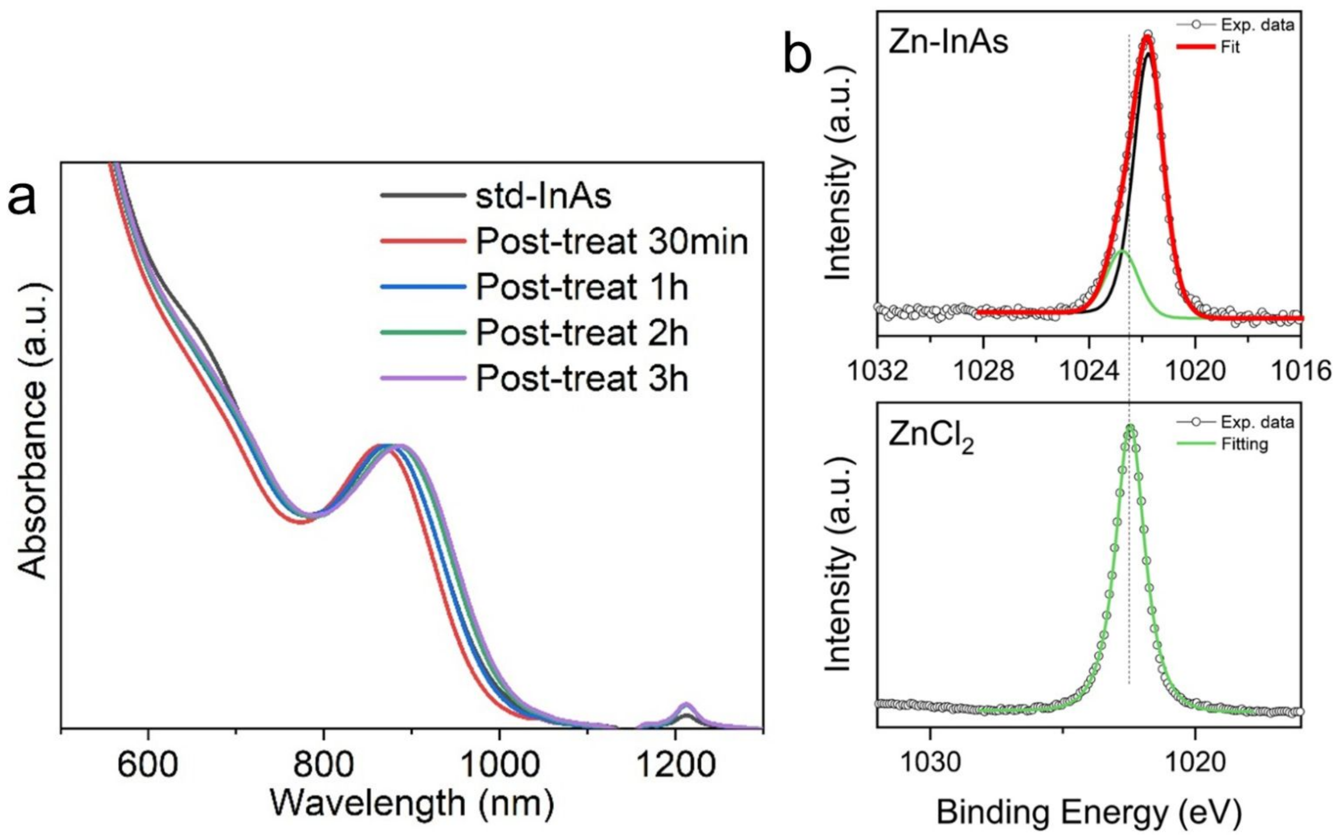

Figure S4. a) Absorption curves of std-InAs QDs subjected to $ZnCl_2$ post synthesis treatment at 280°C for different times. b) XPS spectra of Zn-InAs QDs and $ZnCl_2$ powder in the Zn $2p_{3/2}$ region.

We investigated the effect of air exposure on Zn-InAs QDs by oxidizing them in air for 6 hours and subsequently measuring their XPS spectrum. We observed severe oxidation of As, evidenced by the appearance of new As peaks at 41.2, 43.8 and 45.1 eV, the latter compatible with the presence of As_2O_3, as well as oxidation of In, which resulted in a broadening of the In peak (with the presence of a new component at 445.0 eV compatible with the presence of In_2O_3) (**Figure S5**).[1-3] Additionally, the Zn 2p peak evolved, shifting toward higher binding energies, namely 1022.1 ± 0.2 eV (**Figure S5**). The binding energy of Zn in $ZnCl_2$ (**Figure S4**), compared to that of “oxidized” Zn (**Figure S5**), is closer to the second Zn component (green curve) exhibited by Zn-InAs QDs, further supporting that such component can be ascribed to $ZnCl_2$ species. Moreover, if an oxidation had occurred during the post-synthesis $ZnCl_2$ treatment, that would have caused significant changes in the As and In signals (the majority elements), which however were not observed in the Zn-InAs QDs.

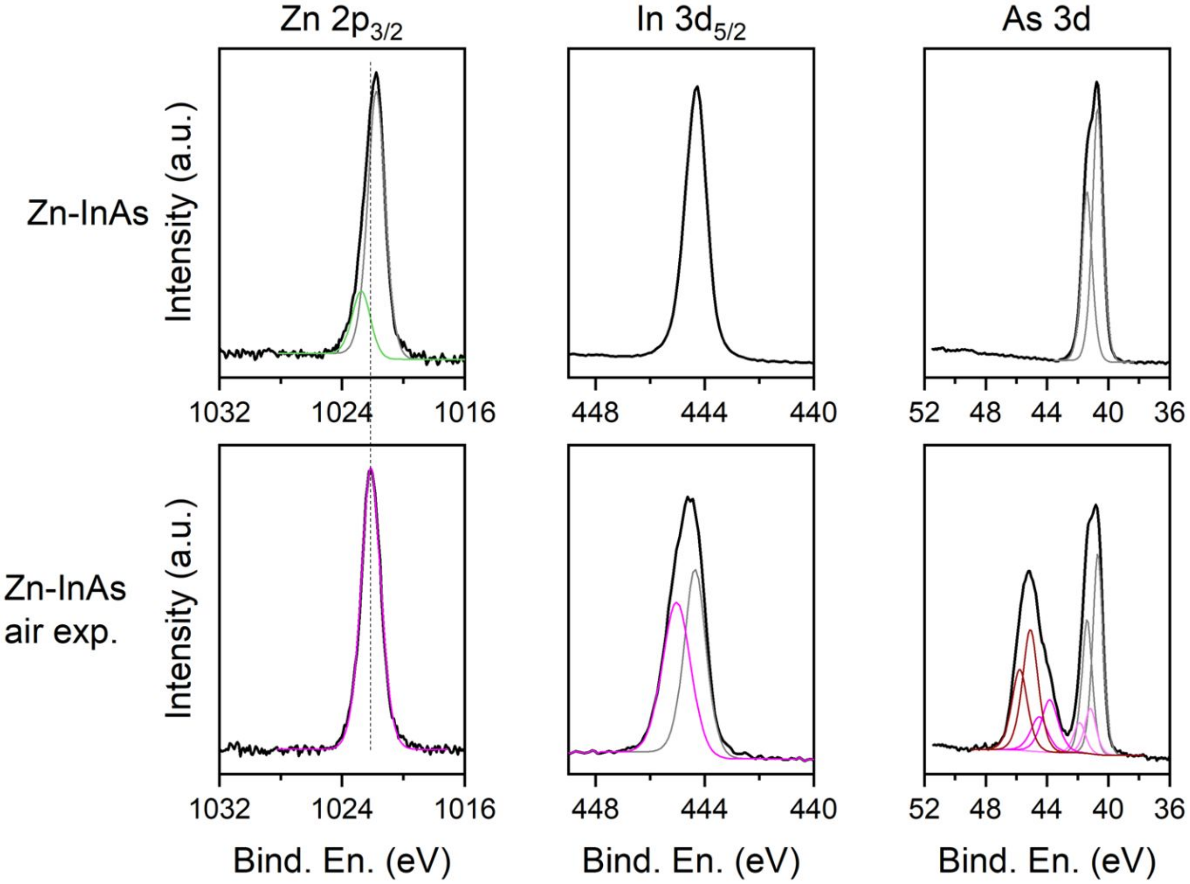

Figure S5. XPS spectra of Zn-InAs QDs in the Zn $2p_{3/2}$, In $3d_{5/2}$ and As 3d regions before and after exposure to air.

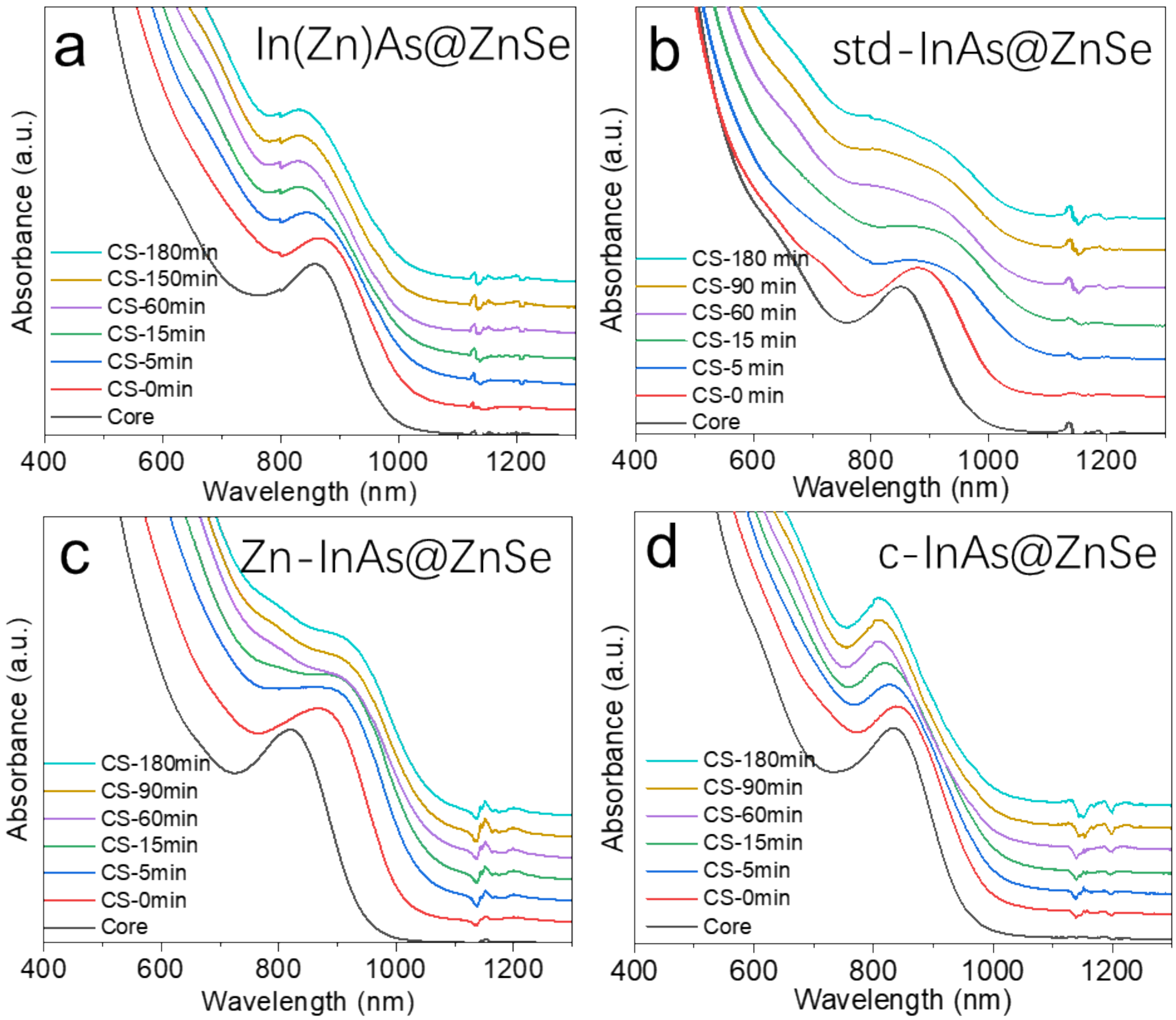

Figure S6. Evolution of the absorption curves of (a) In(Zn)As, (b) std-InAs, (c) Zn-InAs and (b) c-InAs QD samples upon ZnSe shelling from 0 min (the time at which the reaction mixture reaches 300°C) to 180 min. In each graph the black curve named “core” refers to the starting unshelled InAs QDs and CS refers to core@shell samples.

Table S1. Composition of In(Zn)As@ZnSe, c-InAs@ZnSe, std-InAs@ZnSe and Zn-InAs@ZnSe core@shell samples during ZnSe shelling measured via ICP-OES

Sample	ZnSe reaction time	Elemental ratios		
		In/As	Zn/Se	Zn/As
In(Zn)As@ZnSe	Core QDs	1.07	/	0.12
	0 min	1.35	0.83	1.56
	5 min	1.51	0.95	5.66
	15 min	1.58	1	11.28
	60 min	1.76	1	21.36
	150 min	1.84	1	23.71
	180 min	1.89	1	24.39
Std-InAs@ZnSe	Core QDs	1.11	/	/
	0 min	1.42	0.71	0.76
	5 min	1.56	0.87	2.61
	15 min	1.75	0.95	6.13
	60 min	2.01	1.02	21.39
	90 min	2.03	1.02	24.85
	180 min	2.14	1.02	29.71

Sample	ZnSe reaction time	Elemental ratios		
		In/As	Zn/Se	Zn/As
Zn-InAs@ZnSe	Core QDs	1.07	/	0.15
	0 min	1.48	1.16	2.61
	5 min	1.58	1.06	5.81
	15 min	1.65	1.07	9.45
	60 min	1.73	1.05	17.53
	90 min	1.77	1.06	19.53
	180 min	1.76	1.05	20.06
c-InAs@ZnSe	Core QDs	1.07	/	0.12
	0 min	1.42	0.94	1.24
	5 min	1.48	1.04	3.89
	15 min	1.6	1.05	8.08
	60 min	1.77	1.05	21.78
	90 min	1.83	1.07	24.85
	180 min	1.99	1.06	29.01

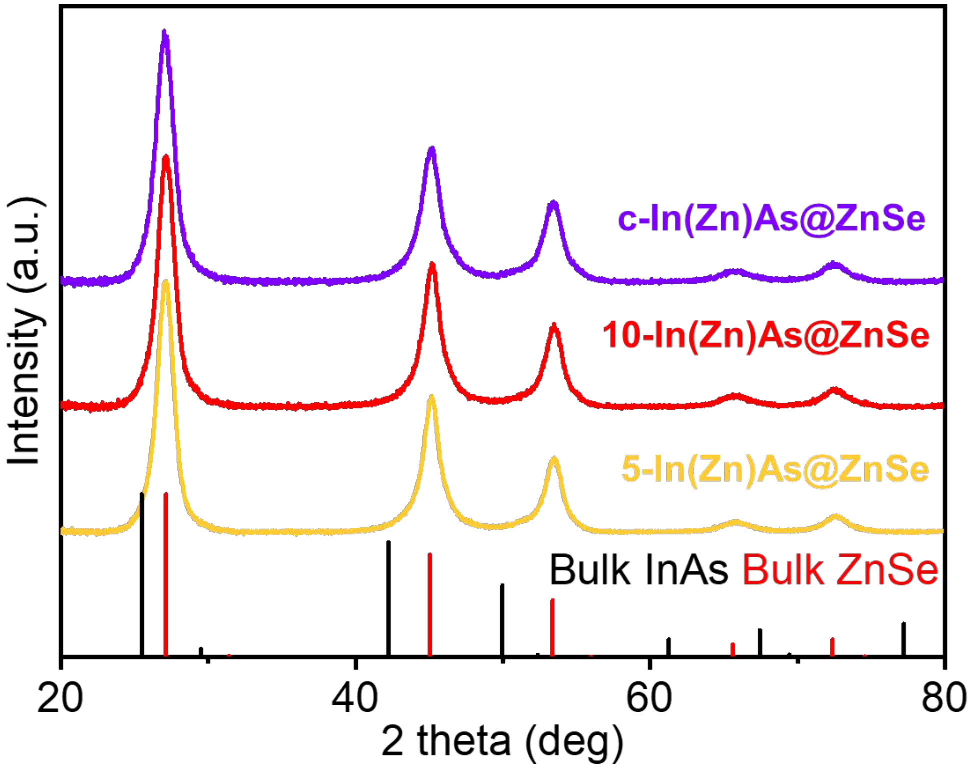

Figure S7. XRD patterns of c-In(Zn)As@ZnSe, 10-In(Zn)As@ZnSe and 5-In(Zn)As@ZnSe QDs with the bulk reflections of InAs (ICSD 98-002-4518) and ZnSe (ICSD 98-007-7092).

Simulation Section

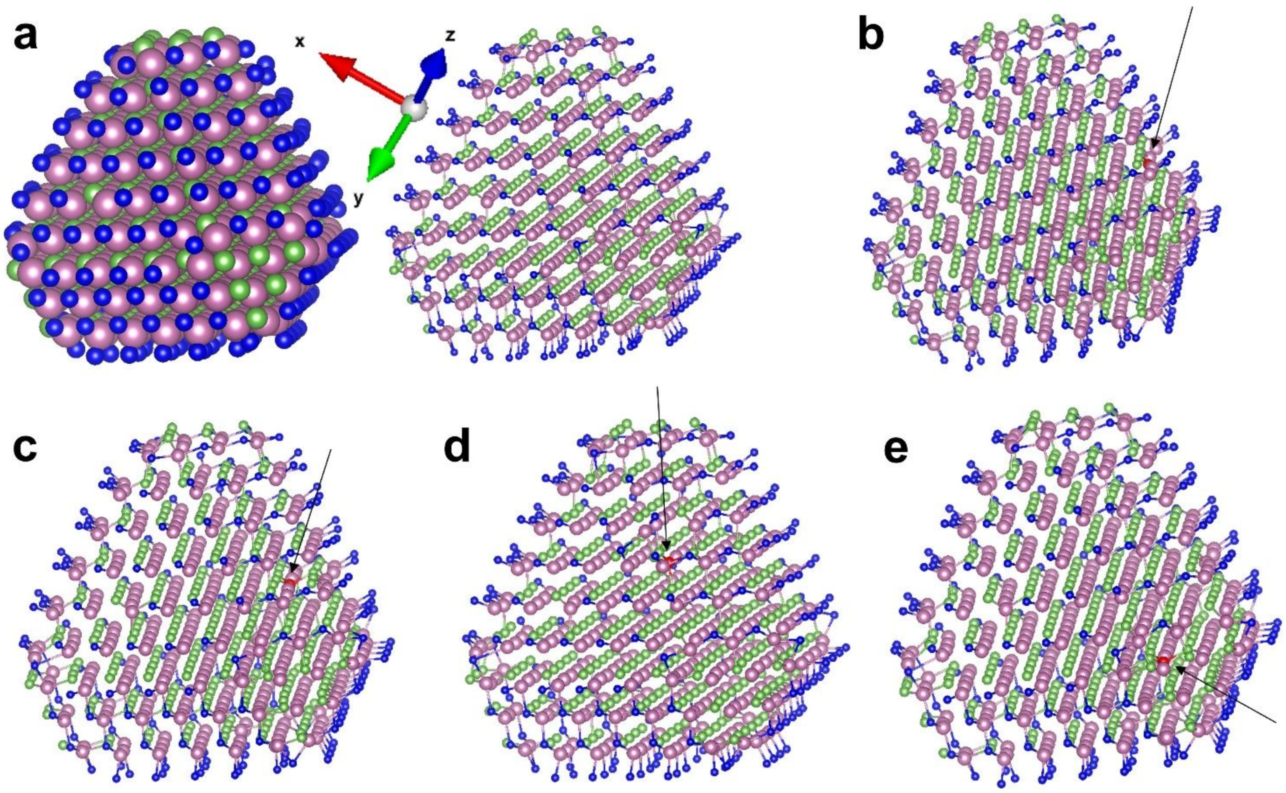

Figure S8. Atomistic models to study the energetics of Zn incorporation within InAs QDs. The basic InAs model is shown in panel (a), in both 'space filling' and 'ball-and-stick' representations, and it was built as follows. A truncated tetrahedron QD of InAs was constructed by cutting bulk InAs along the [111], [100], and [-1-1-1] facets, which form, respectively, the faces, edges, and truncated vertices of the tetrahedral nanocrystal. As the faces and edges are In-terminated, there is an excess of In (in agreement with the experimental observation of the elemental analysis). The QD was charge-balanced by passivating faces and vertices with Cl atoms, in line with experimental composition. In the model of panel (b), a In-Cl fragment is substituted by Zn, in the location where it is most energetically favorable to do so: the surface of a [111] facet. This model is used in **Table S1** as reference. In panels (c), (d), and (e), Zn is put on the subsurface position (2nd layer) of, respectively, a face [111], edge [100], and truncated vertex [-1-1-1] of the QD. In=violet, As=green, Cl=blue, Zn=red. The position of Zn is highlighted by an arrow. These simulations were performed with the same settings as for those discussed in the main text *(e.g.* **Figure 4a**) except that the PBE functional was adopted.[4]

Table S2. Energy difference among the various locations where Zn substitutes In in the InAs QD, see Figure S7

model	Figure	E-E^{reference} (kcal/mol)	E-E^{reference} (eV)
Zn on face	S6b	0.0	0.00
Zn 2nd layer, face	S6c	5.2	0.22
Zn 2nd layer, edge	S6d	23.7	1.03
Zn 2nd layer, vertex	S6e	20.6	0.89

Table S3. Theoretical atomic ratios of the models used.

Model	In	As	Zn	In/As ratio	Zn/As ratio	Zn/In ratio
InAs w/o Zn	780	691	0	1.128	0	0
InZnAs-(100)	770	663	18	1.161	0.027	0.023
InZnAs-(100)+(111)	746	663	42	1.125	0.063	0.056
InZnAs-(111)	746	663	24	1.125	0.036	0.032
InZnAs-(100)+(-1-1-1)	770	671	26	1.148	0.039	0.034
InZnAs-(-1-1-1)	770	671	8	1.148	0.012	0.010
InZnAs-(111)+(-1-1-1)	746	671	32	1.112	0.048	0.043
$ZnCl_2$ (-1-1-1)-ads	780	691	24	1.128	0.034	0.030

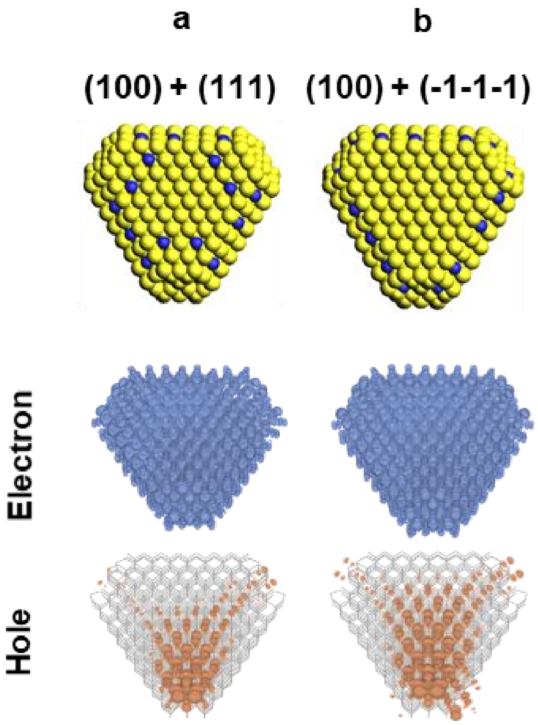

Figure S9. Carriers distribution upon placing Zn atoms on either a) (100) + (111) or b) (100) + (-1-1-1) facets, respectively. Top row: model configurations (yellow = InAs, blue = Zn). Ligands are omitted for clarity. Middle and bottom rows: electron and hole charge densities.

The ATHENA software[5] was used for normalizing the XANES and the EXAFS spectra from the raw absorption data. Pre-edge removal and normalization were achieved using linear functions. The energy threshold (E_0) values were chosen as the first inflection point of the first derivative relatively to the incident energy, and found equals to 9661.3(5) eV and 9661.7(5) eV for In(Zn)As and Zn-InAs respectively. Self-absorption was found negligible from the available tools on the ATHENA software, and therefore no correction has been applied.

The EXAFS data fitting process has been performed by using the ARTEMIS software.[5] Experimental EXAFS spectra were Fourier transformed using a Kaiser-Bessel window over the full k-range available, i.e. 3.538-13.142 $Å^{-1}$. Phases and amplitudes of the interatomic scattering paths were calculated with the ab initio code FEFF8.40.

Four different Zn local environments were considered to fit the experimental spectra, namely Zn atoms incorporated in the (100), (111) and (-1-1-1) facets, and Zn adsorbed as $ZnCl_2$ onto the (-1 -1 -1) facet. This choice was driven by the fact that each facet was giving very distinctive XANES spectra and thus different local environment for Zn. Moreover, the DFT calculations demonstrated the potential role of the preferential incorporation of Zn into specific facets, and EXAFS fitting was used to inform about this hypothesis. We extracted the coordination shell of all 50 Zn atoms included in the DFT model and averaged those coordination shells up to 3 Å (**Table S3** for corresponding shells). Hydrogen atoms were neglected.

Taking all considered facets into account would have exceeded the number of independent parameters to use in the EXAFS fitting process. A simplified model was thus required for EXAFS fitting, and was based on only Zn-Cl and Zn-As scattering paths. This model excludes de facto the possibility of quantitatively assessing the concentration of Zn in each incorporation site, but, thanks to the differences in coordination numbers and distances expected for each occupation site, changes in fitted amplitudes, distances and Debye-Waller factor would infer about the differences between the Zn local environment in Zn-InAs and In(Zn)As samples.

During the EXAFS fitting, the shift in the threshold energy (ΔE_0) was varied as a global parameter and found to be equal to 8.5(4) eV. The product of the scattering factors S_0^2 and the coordination number deduced from DFT was considered as a unique fitting parameter owing their strong dependencies and the limited energy/wave-vector range at our disposal. This is not a limiting factor when comparing the two samples extracted values, but no quantitative analysis of the coordination number can be performed without evaluating the scattering factor. Nevertheless, differences between extracted structural values are already informative enough to assess the Zn local environment differences between both studied samples.

Table S4. Zn 1st and 2nd coordination shells as deduced from DFT calculations as a function of the occupied facet. The asterisk indicates the adsorption of $ZnCl_2$ onto the (-1-1-1) facet. Due to the limited number of path that could be used to fit the available data energy range, only the path in bold where considered to avoid overfitting.

Occupied facet	1st shell	2nd shell
(100)	**2 Cl @ 2.30 Å**	**3 As @ 2.45 Å**
(111)	1 N @ 2.10 Å	**3 As @ 2.50 Å**
(-1-1-1)	**2 As @ 2.30 Å**	1 Cl @ 2.80 Å
(-1-1-1)*	**2 Cl @ 2.13 Å**	**1 As @ 2.45 Å**

References

(1) Song, J. H.; Choi, H.; Pham, H. T.; Jeong, S. Energy Level Tuned Indium Arsenide Colloidal Quantum Dot Films for Efficient Photovoltaics. *Nat. Commun.* **2018**, *9*, 4267, DOI: 10.1038/s41467-018-06399-4.
(2) Ban, H. W.; Vafaie, M.; Levina, L.; Xia, P.; Imran, M.; Liu, Y.; Najarian, A. M.; Sargent, E. H. Resurfacing of Inas Colloidal Quantum Dots Equalizes Photodetector Performance across Synthetic Routes. *J. Am. Chem. Soc.* **2024**, *146*, 24935-24944, DOI: 10.1021/jacs.4c06202.
(3) Henderson, Jeffrey D.; Pearson, L.; Nie, H.-Y.; Biesinger, Mark C. X-Ray Photoelectron Spectroscopy Analysis of Indium and Indium-Containing Compounds. *Surf. Interface Anal.* **2025**, *57*, 81-97, DOI:10.1002/sia.7356.
(4) Perdew, J. P.; Burke, K.; Ernzerhof, M. Generalized Gradient Approximation Made Simple. *Phys. Rev. Lett.* **1996**, *77*, 3865-3868, DOI: 10.1103/PhysRevLett.77.3865.
(5) Ravel, B.; Newville, M. Athena, Artemis, Hephaestus: Data Analysis for X-Ray Absorption Spectroscopy Using Ifeffit. *J. Synchrotron Radiat.* **2005**, *12*, 537-541, DOI: doi:10.1107/S0909049505012719.
